# Genome-Wide Association between Branch Point Properties and Alternative Splicing

**DOI:** 10.1371/journal.pcbi.1001016

**Published:** 2010-11-24

**Authors:** André Corvelo, Martina Hallegger, Christopher W. J. Smith, Eduardo Eyras

**Affiliations:** 1 Computational Genomics, Universitat Pompeu Fabra, Barcelona, Spain; 2 Graduate Program in Areas of Basic and Applied Biology, Universidade do Porto, Porto, Portugal; 3 Department of Biochemistry, University of Cambridge, Cambridge, United Kingdom; 4 Catalan Institution for Research and Advanced Studies, Barcelona, Spain; University of British Columbia, Canada

## Abstract

The branch point (BP) is one of the three obligatory signals required for pre-mRNA splicing. In mammals, the degeneracy of the motif combined with the lack of a large set of experimentally verified BPs complicates the task of modeling it *in silico*, and therefore of predicting the location of natural BPs. Consequently, BPs have been disregarded in a considerable fraction of the genome-wide studies on the regulation of splicing in mammals. We present a new computational approach for mammalian BP prediction. Using sequence conservation and positional bias we obtained a set of motifs with good agreement with U2 snRNA binding stability. Using a Support Vector Machine algorithm, we created a model complemented with polypyrimidine tract features, which considerably improves the prediction accuracy over previously published methods. Applying our algorithm to human introns, we show that BP position is highly dependent on the presence of AG dinucleotides in the 3′ end of introns, with distance to the 3′ splice site and BP strength strongly correlating with alternative splicing. Furthermore, experimental BP mapping for five exons preceded by long AG-dinucleotide exclusion zones revealed that, for a given intron, more than one BP can be chosen throughout the course of splicing. Finally, the comparison between exons of different evolutionary ages and pseudo exons suggests a key role of the BP in the pathway of exon creation in human. Our computational and experimental analyses suggest that BP recognition is more flexible than previously assumed, and it appears highly dependent on the presence of downstream polypyrimidine tracts. The reported association between BP features and the splicing outcome suggests that this, so far disregarded but yet crucial, element buries information that can complement current acceptor site models.

## Author Summary

From transcription to translation, the events underlying protein production from DNA sequence are paramount to all aspects of cellular function. Pre-mRNAs in eukaryotes undergo several processing steps prior to their export to the cytoplasm. Among these, splicing – the process of intron removal and exon ligation – has been shown to play a central role in the regulation of gene expression. It has been estimated that more than half of the disease-causing mutations in humans do so by interfering with splicing. The difficulty in describing these disease mechanisms often lies in the low accuracy of the methods for prediction of functional splicing signals in the pre-mRNA. This is especially the case of the branch point, mainly due to its high sequence variability. We have developed a methodology for mammalian branch point prediction based on a machine-learning algorithm, which shows improved accuracy over previous published methods. Moreover, using a combination of experimental and bioinformatics approaches, we uncovered important positional properties of the branch point and shed new light on how some of its features may contribute to the final splicing outcome. These findings might prove useful for a better understanding of how splicing-associated mutations can lead to disease.

## Introduction

Pre-mRNA splicing, which is essential for the production of functional mRNAs, is a co-transcriptional set of reactions catalyzed by a large ribonucleoprotein complex – the spliceosome – composed by five small nuclear RNAs (snRNAs) and more than hundred proteins [1], [2]. In addition to these core factors, splicing is often dependent on other proteins that can either activate or repress signal recognition, therefore playing a very important role in the regulation of specific events [3], [4]. In a process called Alternative Splicing (AS) introns can be differentially removed, generating multiple isoforms from the same pre-mRNA molecule [5], which is key for the increased protein diversity observed in metazoans [6], [7]. The importance of splicing in the regulation of gene expression is also underlined by the fact that mutations affecting it are frequently associated with, or directly responsible for, severe genetic diseases [8], [9].

**Figure 1:**
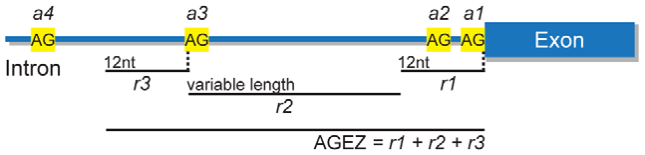
AGEZ definition and BP search region. BP location relative to the 3SS (*a1*) is dependent on presence/absence additional AG dinucleotides in the intron. The most common situation is the absence of AGs in the region between the BP and the 3SS. However, these can occur either at locations close to the 3SS (i.e. *a2* in *r1*) where they may compete with the 3SS signal, or very close to the BP (i.e *a3* in *r3*), where they are bypassed possibly due to steric constraints. Any AG occurring in *r2* is likely to be recognized as 3SS. Therefore, BPs are usually located inside region defined as *r1*+*r2*+*r3* – the AG exclusion zone (AGEZ).

Splicing requires the presence of three main signals that directly participate in the reaction and that are present in every intron: the 5′ splice site (5SS); the 3′ splice site (3SS); and the branch point (BP) [10]. These signals, along with the polypyrimidine tract (PPT), are critical for correct spliceosome assembly [10], [11]. Additionally, there are also *cis*-acting splicing regulatory sequences that can function as enhancers or silencers of splicing [12]–[17]. These are not only important in the regulation of splicing in a context dependent manner, but are also crucial for splice site recognition in general [18]. While the 5SS is located at the start of the intron, the other three core elements – the BP, the PPT and the 3SS – are normally arranged in this order within the last 40 nucleotides (nts). However, this arrangement is not mandatory. There are introns in which the BP can be located up to 400 nts away from the 3SS [19]–[24]. These are referred to as distant BPs (dBPs) and account for approximately 1% of all human introns. dBPs are rarely found by computational methods, since most BP prediction methods use, as condition, the proximity to the 3SS. dBPs have typically an adjacent long PPT downstream and have been associated with AS, in particular with mutually exclusive exons [21], [23]. For both distant and proximal BPs, the region between the BP and the 3SS is usually devoid of AG dinucleotides [22] (Figure 1). However, the AG dinucleotides can either occur at downstream locations close to the BP (distance<12–15 nts – region *r3* in Figure 1), where they can be bypassed, or close to the 3SS (distance<12 nts – region *r1* in Figure 1), where they would be competing with the actual 3SS [25]. This extended region is named AG Exclusion Zone (AGEZ).

In mammals, while both 5SS and 3SS signals have been mapped precisely by aligning transcriptional evidence to the genome, allowing the development of robust statistical models from large datasets [26], [27], BP characterization has been a far more complicated task. Firstly, the lack of a sufficiently large “gold standard” set of mammalian BPs – only a few dozens have been mapped [28], [29] – makes difficult the task of building statistical models. Additionally, unlike for some fungal species, which present very strict BP consensus [30], [31], the mammalian BP is an extremely degenerate motif [28]. This is attested to by recent studies that had focused on the BP signal and on its relation with splicing factors across a wide range of species, including mammals [32], [33]. These studies based their BP predictions on the Hamming distance to the U2 complementary sequence TACTAACAC [33]. While such approach has been used successfully in fungal species, it proves insufficient for mammals where the resulting motif consensus reflects, above all, the background nucleotide frequencies and the consensus used to search it.

In this paper we present a new strategy for predicting BPs in human. Using conservation and positional bias we first built a set of high-confidence putative BPs. This set was then used as a positive training set for a Support Vector Machine (SVM) learning algorithm that combined both BP and PPT information into a predictive model. We show that this method outperforms previously published methods for both proximal and distant BPs. Applying our predictive algorithm to human introns, we are able to characterize the localization of the BP within the intron and describe how BP signal features may contribute to the final splicing outcome. Moreover, we experimentally determined the BP location for some introns containing long AGEZs, which are characterized by the presence of dBPs, and in which we observed alternative BP usage. Our computational and experimental analyses suggest that BP recognition is highly plastic, partially dependent on downstream PPT features, and of critical importance to the final outcome of the splicing reaction.

## Results

### Reference BP set

**Figure 2:**
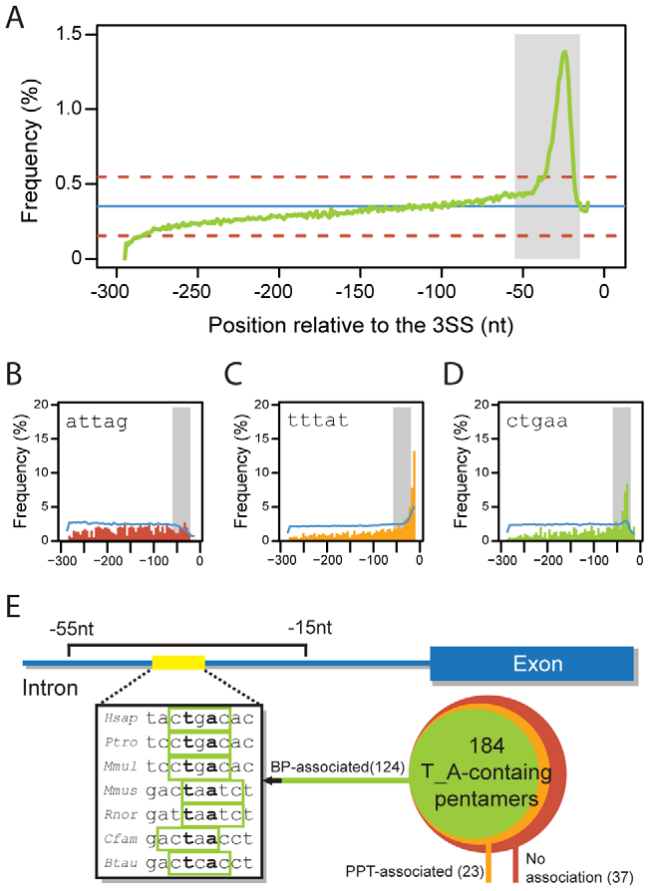
Building a set of conserved putative BPs. **A** – Distribution of mammalian wide conserved TNA instances in the last 300nt of human introns. The blue line represents the mean frequency. The dashed red lines represent the mean+- the standard deviation. The grey area represents the region comprehended between 55 to 15 nt upstream the 3SS. **B**,**C** and **D** – Distribution over the last 300 nts in human introns for the mammalian-conserved instances of 3 example pentamers belonging to different categories: No association with any positionally biased signal (B), PPT-associated (C) and BP-associated (D). The line in blue represents the distribution of all (conserved and non-conserved) instances. The grey area represents the region comprehended between 55 to 15 nt upstream the 3SS. **E** – Scheme representing the employed strategy to build a set of conserved putative BPs. We selected TNA conserved instances located between 55 and 15 nts upstream the 3SS and unique to the last 300nt of the intron, if overlapped by at least one BP-associated pentamer in all species considered (see Methods).

**Figure 3:**
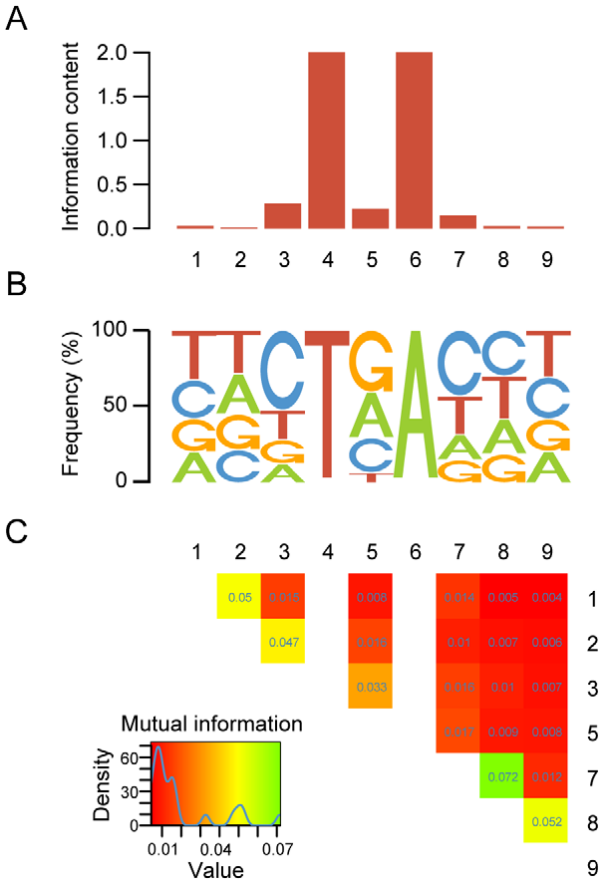
BP signal characterization. **A** – Information content per motif position. Both position 4 and 6 were previously fixed. **B** – Sequence logo for the *consTNA-BP5* set. The height of each letter represents the frequency of that nucleotide in the respective position. **C** – Mutual information between BP signal positions. Blank spaces represent the two invariable positions 4 and 6.

**Figure 4:**
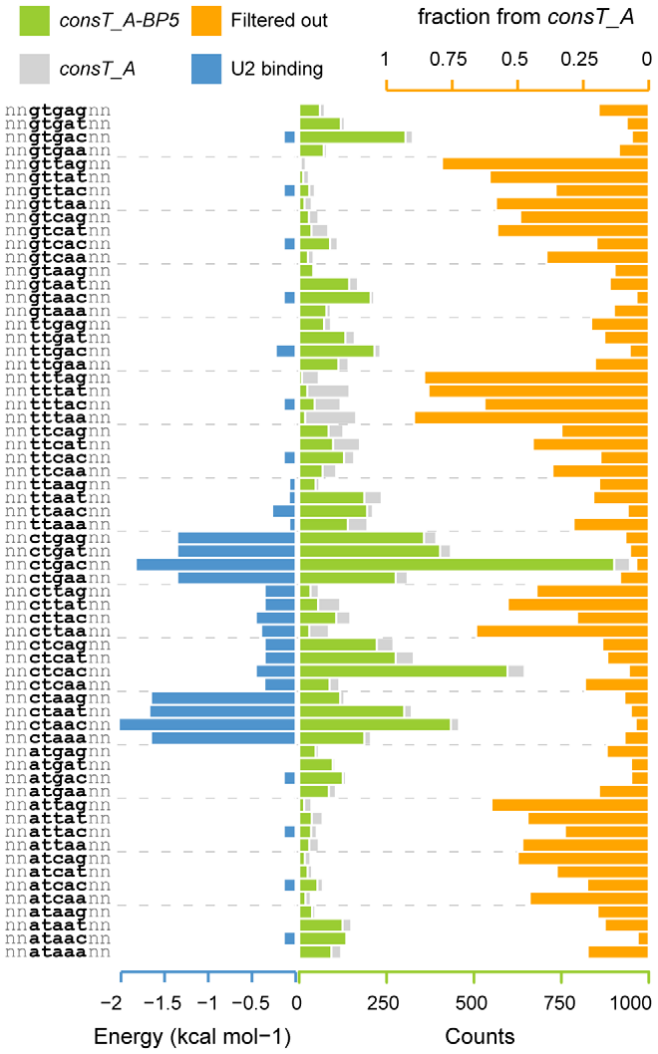
Sequence counts correlate with U2 binding energy. Barplot showing, for each nonamer cluster, the U2 binding energy (blue), number of occurrences in the *consTNA* and *consTNA-BP5* sets (grey and green, respectively). The fraction of eliminated cases by the use of the 124 BP-associated pentamers is also shown in orange. Nonamer clusters were grouped by core pentamer (5 central positions).

To circumvent the lack of a large set of experimentally verified BPs from which a predictive model could be derived, we decided to build a set of high-confidence putative BPs and use it as positive training set. Rather than using as starting hypothesis a strict consensus and finding sequences that are similar to it, we tried to capture an unbiased BP sequence signal using positional and conservation principles. The mammalian BP is a quite degenerate motif with only two highly constrained positions, the branch point A and a T two bases upstream, which we denote as TNA. If we consider this motif alone, we observe that it is strongly conserved towards the last 50 nt of the introns (Figure 2A), with a peak around position −23 relative to the 3SS (see Figure 1 in Text S1 for all the other trinucleotide combinations). Surprisingly, a simple motif overrepresentation approach does not allow the identification of words that can potentially be associated with the TNA distribution profile. Indeed, if one computes pentamer frequencies in the region spanning from position −55 to −15 relative to the 3SS in human, the large majority of the most abundant pentamers do not contain the TNA motif and appear to be associated with the PPT signal due to their high pyrimidine content (see Table 1 in Text S1). Moreover, very few TNA-containing pentamers present a non-uniform distribution profile over the last 300 nts of human introns, hardly being representative of the expected BP signal variability (Figures 2–4 in Text S1). Thus, we considered a comparative approach. Under the assumption that functional sites are potentially more conserved than non-functional ones, we expect BP-related words to be more conserved in the region for which we observe a peak in the TNA motif distribution. Accordingly, by considering only TNA-containing pentamer instances that were perfectly conserved across 7 mammalian species (*Homo sapiens*, *Pan troglodytes*, *Macaca mulatta*, *Mus musculus*, *Rattus norvegicus*, *Canis familiaris* and *Bos taurus*), we were able to find a clear distribution profile for each pentamer. Next, by performing a set of statistical tests to these profiles (see Methods), we were able to separate the set of all 184 TNA-containing pentamers into 3 categories based on their positional bias: 1) No association with any positionally biased signal (N = 37), 2) PPT-associated (N = 23) and 3) BP-associated (N = 124) (see Methods). Example pentamers for each category are shown in Figures 2B, 2C and 2D (see Figures 2–4 in Text S1 for all 184 TNA-containing pentamers). Finally, to build the final set of putative BPs, we selected all 9-mers including conserved TNA instances in their central position that were unique in the last 300bp of an intron, falling between positions −55 and −15 relative to the 3SS (*consTNA* set – for further reference). This set was subsequently filtered, forcing the overlap of at least one BP-associated pentamer in each species. In this manner, we allow sequence variability maintaining functional conservation (Figure 2E). After this step we were left with a set of 8156 conserved putative BPs, which we denote as *consTNA-BP5* (provided in Dataset S1).

### Signal characterization

Using the *consTNA-BP5* set, we derived the sequence logo corresponding to the BP signal in human (Figure 3B). In accordance with previously published studies, the human BP signal is quite variable, presenting very low information content (IC = 4.739, including the fixed T and A positions) (Figure 3A). Moreover, our results corroborate the YTNAY consensus determined experimentally in [28]. Nevertheless, there seem to be some constraints on the central position, where T appears at a very low frequency, while G, A and C are found with much higher probability (Figure 3B). Positions +1 and −3 relative to the BP A seem also to contain more frequently purines than previously assumed. In order to address whether specific nucleotide combinations are more or less frequent than expected under a model of independence between positions, we used the Mutual Information (MI) measure (see Methods). We found dependencies between adjacent positions in the BP signal (discarding the 2 fixed positions) (Figure 3C). Moreover, we also found weak second-order dependencies involving the central positions of the signal. Although MI values are low in general, the relatively large set size allows us to capture these small dependencies, which we use to describe the sequence signal with a position-dependent Markov model of order 1, which we denote as MM1 (see Methods).

### Putative BP signal correlates with U2 binding energy

**Figure 5:**
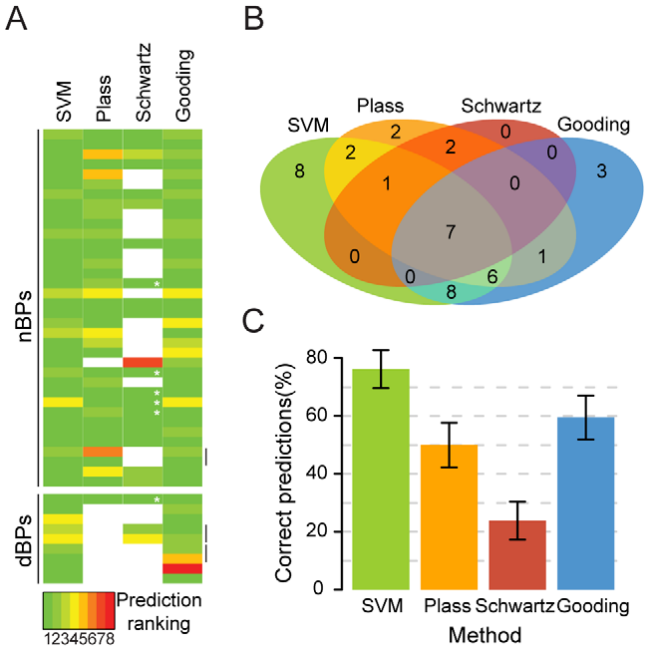
Benchmarking on a set of experimentally verified BPs. **A** – Ranking of experimentally verified BPs according to 4 predictive methods. Blank cells represent BPs that either did not match the initial sequence requirements or that are located outside the search region. Though Schwartz method in several introns ranks the BP as 1^st^, the prediction is discarded because it is not the candidate closest to the 3SS (white asterisks). **B** – Correct predictions overlap between methods. **C** – Percentage of introns in which each method was capable of correctly predicting the BP. The error bars represent the standard error given by the formula: 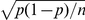 where 
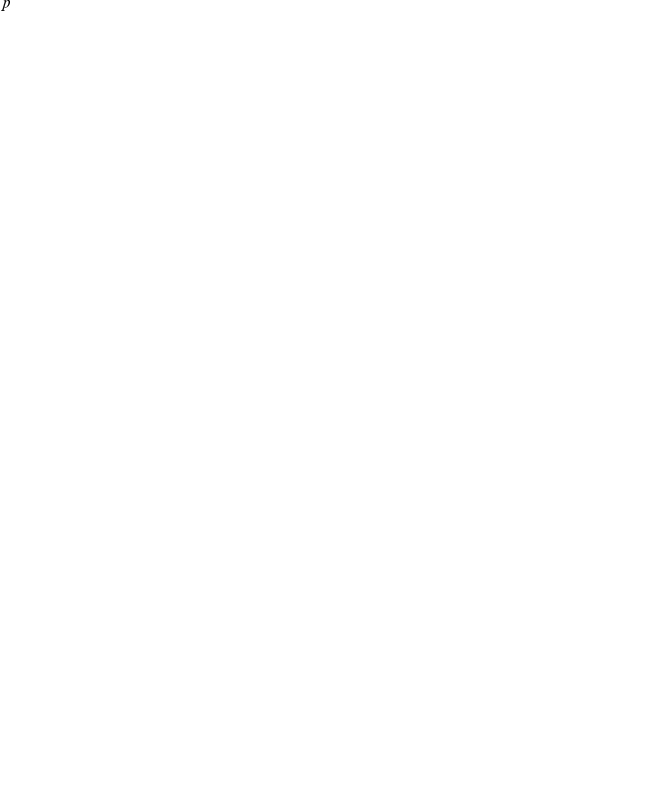
 is the probability and n the overall sample size.

To test whether the frequencies of different BP-associated words correlate with the U2 binding stability, we grouped all the *consTNA-BP5* 9-mers by their central pentamer sequence, which are the five positions with higher IC in the BP signal, and calculated the mean U2 binding energy for each group (see Methods). In Figure 4 we can observe that there is a direct correlation between the stability of the binding to the U2 and the occurrence of these words in the *consTNA-BP5* set (Spearman's rank correlation, rho = −0.65, *p* = 6.53×10^−9^, see Figure 5 in Text S1), which validates the captured BP signal. Interestingly, if we compare this set with the *consTNA* set, we observe for the *consTNA-BP5* a drastic reduction in the frequency of some words for which the U2 binding energy is low (Figure 4). In fact, these are mainly PPT-associated words which were filtered out from the *consTNA* set. Thus, we can consider the *consTNA-BP5* set as a good representative of putative functional BPs.

### A predictive model for BPs

**Figure 6:**
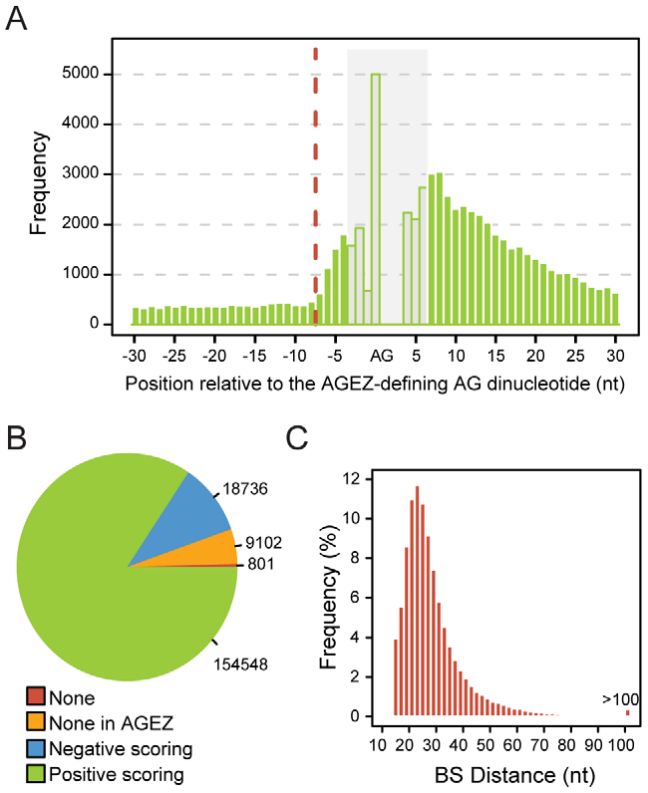
Predicted human branch points. **A** – Histogram representing the distribution of BS positions relative to the AGEZ-defining AG-dinucleotide (*a3* in Figure 1). Grey region represents positions that are biased by the presence of the AG dinucleotide. The dashed red line represents the leftmost point where the distribution is different from an expected uniform distribution. The AG dinucleotide exact position is shown on the x-axis. For this plot, top scoring candidates over the last 500nt were considered in order to obtain the left background tail. For visualization purposes only positions from −30 to +30 nts relative to the AG are shown. **B** – Pie chart showing the number of introns in the initial dataset (N = 183187) for which no predictions were obtained (None), no predictions falling inside the 1^st^ AGEZ were obtained (None in AGEZ), the top prediction inside 1^st^ AGEZ has a negative SVM score (Negative scoring) and the top prediction inside the 1^st^ AGEZ scores positively (Positive scoring). **C** – Histogram showing the distribution of predicted BS distances relative to the 3SS. Only top scoring candidates inside the AGEZ were considered.

The low IC observed for the BP signal motivates the addition of other sequence features in a predictive model. We therefore considered a model that incorporates the relation between the BP and the properties of downstream proximal PPTs. We took as positive training set the *consTNA-BP5* set and as negative a random set of intronic 9-mers containing TNA in the same position (see Methods). For every candidate BP, from both positive and control sets, we computed the sequence score with a position-dependent Markov model of order 1 (MM1) and three PPT-related features (see Methods). These features were used as input for an SVM learning algorithm, which produces a score that reflects the distance in feature space between the candidate and the decision boundary. Elements for which the SVM score is above the threshold (zero, in this case) are labeled as positive, while elements scoring below the threshold are considered negative. The higher the score is (in absolute terms), the more reliable the prediction. This method shows good discriminative power between positive and negative BP candidates. Indeed, using a 10-fold cross-validation, the average accuracy for our method is 0.794±0.011 and the receiver operator characteristic (ROC) analysis shows an area under the curve (AUC) of 0.878±0.011. Additionally, in order to understand the effect of incorporating PPT information in the model, the SVM model was compared with the position-dependent Markov model MM1 and a position weight matrix (PWM) model. We show in Figures 6A and 6B in Text S1 that the SVM model outperforms the other two methods (AUC, MM1: 0.778±0.013 and PWM: 0.764±0.014) not only in accuracy but also in precision. This difference reflects the importance of additional sequence features in BP recognition. Relative to the comparison between MM1 and PWM, Figure 6 in Text S1 shows that incorporating dependencies between positions yields extra, though marginal, predictive power.

### Benchmarking on a set of experimentally verified BPs

In order to test our SVM model in a more realistic situation, we compared the predictions on a set of experimentally verified BPs (provided in Dataset S1) with other previously published methods for BP prediction. Thus, we collected a set of 35 human introns that were not part of the training set and for which at least one TNA-containing BP had been experimentally determined. Additionally, 7 introns containing experimentally verified dBPs were added to this test set. Out of the 42 introns, 3 contained more than one mapped BP. For these cases, we considered a prediction as correct if any of them were detected. Using our SVM model, we considered as positive the highest scoring hit falling in the AGEZ. BP search was also performed using 3 other methods from recent publications. Two of these methods, Schwartz [32] and Plass [33] methods are based on the complementarity to the U2. In these two cases, candidates are ranked according to their Hamming distance to a strict consensus. The third method, Gooding method [22] scores candidates using a PWM trained from human data and it has been successfully applied to find dBPs. Additionally, while Plass and Schwartz methods search over a region of length 100 nts and 200 nts respectively, ours and Gooding's methods search candidates in the AGEZ only. Benchmarking results are shown in Figure 5 in terms of sensitivity, computed as the number of true positives (TP) over the total number of introns. Our SVM model shows the best performance, determining correctly the BP for 76% of the introns tested (Figure 5C). Gooding method, also trained on human data, comes up as second best, predicting correctly the BP for approximately 60% of the introns. There is a big overlap between these two methods, with about 84% of Gooding predictions being also predicted by our SVM model (Figure 5B). Hamming distance based methods are the least accurate, in part due to their high strictness. In Figure 5A, blank spaces are BPs that, in each method, either did not match the initial sequence requirements or are outside the search region. Moreover, in Schwartz method, some BPs that are ranked 1^st^ are ultimately discarded because they are not the closest candidate to the 3SS. Consequently, Schwartz method has the highest positive predictive value (PPV) of all four (0.83%). However, sensitivity (0.24%) is negatively affected.

### Experimental BP mapping in introns containing long AGEZs

**Figure 7:**
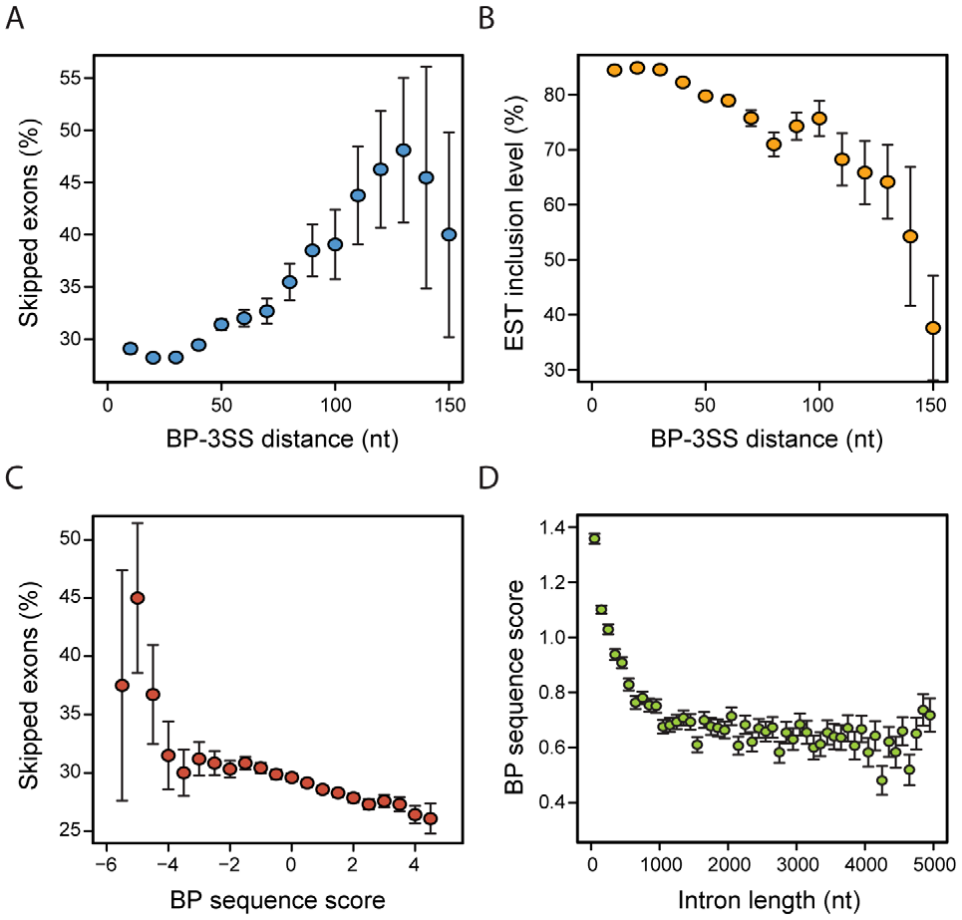
BP sequence, position, intron length and exon skipping. Percentage of exons for which (**A**) there is skipping evidence and (**B**) average exon EST inclusion level depending on BP distance. These values were computed using a sliding window of length 20 and step 10. **C** – Percentage of exons for which there is skipping evidence depending on BP sequence score. This was computed using a sliding window of length 1 and step 0.25. **D** – Mean BP sequence score as a function of intron length. This was computed in bins of 100 nts. The error bars represent the standard error. In A and C, the standard error is given by the formula: 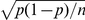 where 
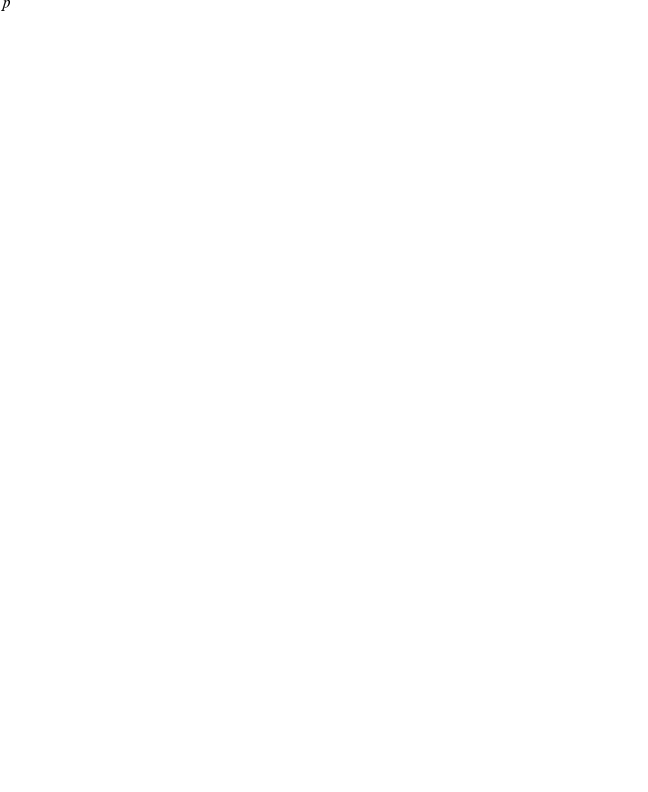
 is the probability and 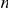 the overall sample size.

**Figure 8:**
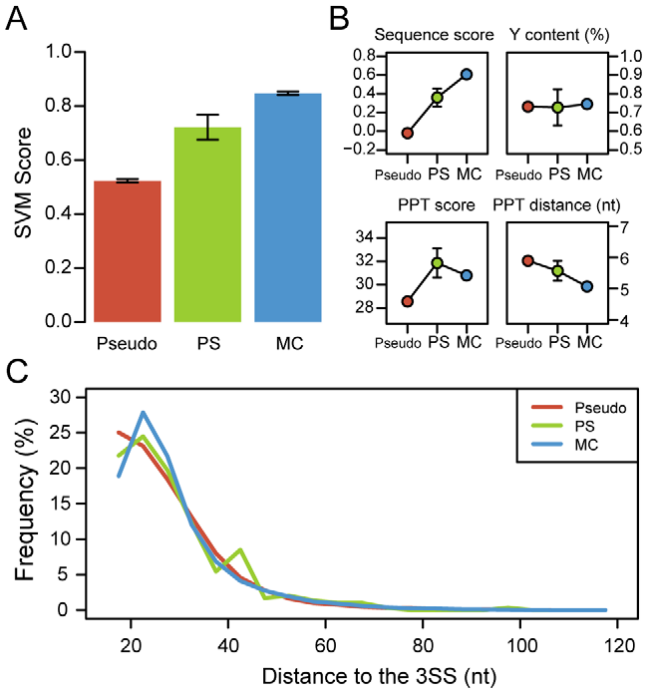
BP features and exon age. **A** – Mean SVM score for BPs preceding pseudo exons, primate specific exons and mammalian-conserved exons. **B** – BP features for the same three exon groups: sequence score (top left), pyrimidine content between BP and 3SS (top right), downstream PPT score (bottom left) and distance to the downstream PPT (bottom right). **C** – BP-3SS distance for exons in the three above mentioned categories.

To further assess the accuracy of our predictive algorithm in the particular case of long AGEZ-containing introns, where potential dBPs might be present, we decided to experimentally determine the BP position for several exons preceded by long AGEZs by means of *in vitro* splicing reactions followed by primer extension. We subsequently compared the mapped BPs to our ranking of candidates given by our SVM algorithm (Figures 7–11 in Text S1). For this analysis, we considered 3 exons from the muscleblind-like 1 (*MBNL1*) gene, which are preceded by long AGEZs (exons 6, 8 and 9, with AGEZs of length 173, 134 and 224 nts, respectively) and exon 4 from two members of the CDC-like kinase protein family (*CLK1* and *CLK3*), both preceded by long AGEZs of 235 and 207 nts in length, respectively. Additionally, 4 exons with long AGEZs from the serotonin receptor 4 (*HTR4*) gene (exons 3, 4, 5 and g, with AGEZs of length 149, 291, 221 and 101 nts, respectively), for which the BP location has been recently determined [34], were added to complement the analysis. Results are summarized in Table 1. With the exception of *MBNL1* exon 8, which is nevertheless characterized by two BPs located at non-canonical positions (−51 and −64), all exons use dBPs, further underlying the association between long AGEZs and the usage of dBPs. Remarkably, in most cases there is not a unique BP, but more complex arrangements where several different BPs, some of which located at a more canonical position, can be alternatively used (Figures 7B, 8B, 9B, and 10B in Text S1). Interestingly, for *MBNL1* exon 9 the predominant BP is at −229. The two very weak signals, located at more canonical positions (−31 and −41), appear to be associated with a slower migrating lariat species that was only observed at the last time point of the *in vitro* splicing reaction (Figure 9A in Text S1), whereas the more rapidly migrating lariat, corresponding to the −229 BP, appeared much earlier.

**Table 1:** BP mapping in long AGEZ-containing introns.

Gene name	Exon/length (nt)	AGEZ length (nt)	Candidates in AGEZ (N)	BP position (nt)	BP sequence	MM1 ranking	SVM ranking
*MBNL1*	6/54	173	11	−141	cgatgAttt	5	2
				−144	ttccgAtga *	-	-
	8/36	134	13	−51	ttttaAttc	6	4
				−64	gtgtgAtgg	3	2
	9/95	224	14	−31	gctacActc *	-	-
				−41	tctgtAtgt *	-	-
				−229	tggtaAcaa	1	1
*CLK1*	4/91	235	17	−224	atctgAaaa	9	4
				−229	atcttAtct	14	10
*CLK3*	4/97	207	11	−196	tcttgAcgt	3	2
*HTR4*	3/201	149	5	−143	atgtgActt	3	1
				−151	cactaAgca	1	2
	4/154	291	11	−27	tgcctAtgc *	-	-
				−33	tgcccAtgc *	-	-
				−72	ctctcAtat	3	5
				−267	taatcActt	4	1
				−273	attatAtaa *	-	-
	5/569	221	10	−26	tcctcAttt	3	9
				−39	tttttAcct	7	10
				−213	tgctgAtaa	2	1
	g/76	101	6	−31	ccctcAtct	2	2
				−86	tactaAtct	1	1

*non-canonical BP.

In 5 out of the 9 introns considered here, our SVM classifier was able to rank as top prediction at least one of the used BPs (Table 1). In three of the remaining cases, at least one of the used BPs in each of the introns ranked second according to the SVM score. The modest performance of the SVM classifier in these cases is in part explained by the generally weak sequence score observed for most of the BPs. However, these weak signals are usually compensated by the presence of a downstream PPT (see Figures 7C, 8C, 10C, and 11C in Text S1), which results in a ranking improvement. This is the case for the *MBNL1* and *CLK* exons, where it is always possible to observe an increase in ranking from the MM1 sequence score to the final SVM score (Table 1). One example is the BP located at position −141 relative to *MBNL1* exon 6. Considering signal strength only, this BP ranks 5^th^ among all the 11 candidates present in the AGEZ. Interestingly, our SVM classifier places it as the second best prediction for that intron, with a resulting score slightly lower than the obtained for a BP candidate located at position −84, also preceding a PPT and for which the sequence signal is considerably stronger (Figure 7C in Text S1). Similar situation can be observed for *MBNL1* exon 8, where a very strong BP signal (tgctgAcag) at position −138 followed by a PPT of considerable length leads to misprediction (Figure 8C in Text S1). Here again, the ranking according to the SVM classifier for both mapped BPs (at positions −64 and −51) is better when compared to the MM1 score (raising from 3^rd^ and 6^th^ to 2^nd^ and 4^th^, respectively). Concerning *CLK1* and *CLK3* exons, in both cases the BPs are located towards the 5′ end of the AGEZ. Interestingly, despite the high pyrimidine content in these regions, there is not a continuous PPT stretch due to frequent purine interruptions (Figures 10C and 11C in Text S1). Prediction in this situation is additionally hindered by the presence of much stronger signals located towards the middle of the AGEZ, which are also associated with PPTs of considerable length. In respect to *HTR4* predictions, our SVM classifier identified as top prediction for exon 3 a BP candidate located at position −143. Despite the fact that primer extension experiments point to the usage of a BP located at position −151 (ranking second according to the SVM classifier), further mutagenesis analyses suggested the possible usage of the first predicted site [34]. Regarding the remaining *HTR4* exons, our SVM algorithm was capable of top ranking each BP mapped by primer extension. It is worth mentioning that all *HTR4* exons considered here are characterized by having the BP localized towards the 5′ end of the AGEZ and by the presence of long PPTs covering almost the totality of the AGEZ.

### Genome-wide BP prediction in human

Using the SVM classifier, all introns in our human dataset (N = 183187) were scanned for BPs. In order to study the relation between the AGEZ and BP position in more detail, all BP candidates falling in the last 500nt of every intron were scored, regardless of being in the AGEZ or not. For introns shorter than 500nt, the entire intron was scanned. In Figure 6A, we plot the distribution of the BP A position of the best hits per intron relative to the AGEZ-defining AG-dinucleotide (*a3* in Figure 1). We observe that the most frequent location of the BP is inside and towards the 5′ end of the AGEZ. The left-most tail in the distribution reflects the background probability of finding a high scoring BP candidate in all the intron. Interestingly, from 5′ to 3′, the frequency of occurrences increases, starting at a distance of 7–8 nucleotides upstream the AGEZ-defining AG-dinucleotide. This distance is shorter than the 12nt considered when defining the AGEZ (see region *r3* in Figure 1). These results suggest that the BP can be most frequently found within the AGEZ and that there is no need to search beyond that. In effect, only in approximately 5% of the introns no candidate was found in the AGEZ. For the remaining 95% we were able to retrieve candidates within the AGEZ, of which approximately 89% score positively (Figure 6B). This percentage drastically drops when considering the next AGEZ upstream of this one (Figure 12A in Text S1), where only in less than 25% of the cases there is a positive hit. When considering the top scoring candidates in the AGEZ (our set of predicted human BPs from this point on), we can observe a distribution bias with approximately 96% of the cases falling between −15 (downstream limit) and −55nt relative to the 3SS with a peak at position −24. However, the distribution extends up to almost the maximum of 500 nt, with ever-diminishing frequencies (Figure 6C). Considering dBPs as predicted BPs that lie beyond 100bp from the 3SS, i.e. 4 times the average 3SS-BP distance, these account for a very small percentage (0.4%, n = 688) of the total predicted BPs (n = 173284). Comparing this set with BPs predicted in the standard range (−55,−15) (Figure 13 in Text S1), we found that dBPs have stronger motif sequences (Mann-Whitney, *p* = 1.34×10^−29^). Interestingly, the pyrimidine content between dBP and the 3SS is similar to closely located BPs (Mann-Whitney, *p* = 0.24), which is surprising considering the large distance. Consequently, PPTs nearby the dBPs are longer and thus have higher score (Mann-Whitney, *p*≈0). In summary, this leads to higher SVM scores for dBPs (Mann-Whitney, *p*≈0).

### BP-3SS distance and BP-sequence are associated with exon skipping

Interestingly, BP-3SS distance positively correlates with AS. Skipped exons tend to be more frequently preceded by introns containing distant BPs than constitutive exons (Mann-Whitney, *p* = 1.97×10^−8^) (Figure 7A). As the BP-3SS distance increases, so does the percentage of exons for which there is skipping evidence. It is possible to observe an almost linear correlation between BP distance and frequency of skipped exons. We found skipping evidence for approximately 43% of the exons in which the BP is located at more than 100 nts upstream, whereas for exons preceded by proximal BPs (3SS-BP distance<50 nts), only 28.6% of them were skipped (Chi-square, *p* = 1.61×10^−6^). Remarkably, this association also holds for the exon inclusion level. For the fraction of skipped exons, inclusion was calculated based on expressed sequence tag (EST) data (see Methods) and is plotted in Figure 7B. Exon inclusion decreases with BP distance. While skipped exons preceded by proximal BPs (distance<50 nts) are included in average in 85% of the transcripts, this value drops down to 65% for exons with a distal BP (3SS-BP distance>100 nts) (Mann-Whitney, *p* = 2.87×10^−9^). Additionally, BP sequence score also correlates with AS. In Figure 7C, we observe that skipping of the downstream exon is more frequent for introns with lower BP sequence score. This increase in skipping is fairly gradual. Even though the sequence score distribution is skewed towards high values (not shown), the difference in skipping percentage between lower and upper sequence score quartiles (defined by scores lower than −0.338 and higher 1.838, respectively) is strongly significant (Chi-square, *p* = 1.83×10^−10^). Moreover, there is small, but statistical significant, difference in BP sequence score between skipped (mean = 0.706) and constitutive (mean = 0.797) exons (Mann-Whitney, *p* = 1.78×10^−9^), further validating that observation.

### Short introns vs. long introns

We also found clear differences in the BP signal between short and long introns. Shorter introns have in general strong BPs (Figure 7D). Indeed, introns of length up to 100 nt, contain BP sequences scoring almost 1.4 in average. This value tends to decrease in a gradual manner as intron length increases up to approximately 1000nt (Spearman's rank correlation, rho = 0.12, *p*≈0), where from that point on it stabilizes (Spearman's rank correlation, rho≈0, *p* = 0.154). Interestingly, even though pyrimidine content between the BP and the 3SS is higher in shorter introns, the overall PPT score is lower, possibly due to slightly shorter PPTs (see Figure 14 in Text S1). Nevertheless, the final SVM score for shorter introns is higher (Figure 15A in Text S1). Another interesting observation is that shorter introns have, in average, lower BP candidate density, both in the AGEZ, or when considering the last 100 nts (Figure 15B in Text S1). This fact cannot be explained by the shorter AGEZs in short introns, as differences are small.

### BPs and exon age

Recent studies have reported a strong relation between exon age and AS [18], [35], [36]. It has been suggested that the low inclusion observed for young exons is due to weaker splicing signals in general [18]. In order to investigate whether BP features are also related to the differences observed between exons with different evolutionary age, we predicted BPs in three exon sets: primate specific (PS) exons; mammalian conserved (MC) exons; and, as control, a set of pseudo exons, which have no inclusion evidence. In Figure 8A we show that BPs preceding real exons have in average higher SVM scores than those preceding pseudo exons (Mann-Whitney, *p*≈0). This difference is even greater when comparing to BP candidates preceding random AG dinucleotides (Figures 12B, 12C in Text S1), since pseudo exons are preceded by a PPT signal, contributing to a higher BP SVM score (see [18] for details on the pseudo exon set construction). Interestingly, BP preceding PS exons, which have low inclusion, have intermediate values between pseudo exons (Mann-Whitney, *p* = 6.18×10^−5^) and MC exons (Mann-Whitney, *p* = 0.022). As we show in Figure 8B, these differences are mainly explained by differences in sequence score (Mann-Whitney, pseudo vs. PS *p* = 7.58×10^−5^; PS vs. MC *p* = 0.039). Regarding intronic position, BPs preceding pseudo exons tend to be located closer to the 3SS compared to real exons (Mann-Whitney, *p*≈0). BPs preceding real exons show a distribution peak between positions 20 to 25 nts upstream of the 3SS, whereas in pseudo exons this peak is located at the smallest distance considered (15 nts). Finally, no differences were found between PS and MC exons (Figure 8C) regarding BP-3SS distance. This feature strongly correlates with AGEZ length, which does not differ significantly between sets (data not shown).

## Discussion

### Signal identification and characterization

Perhaps the major challenge in mammalian BP prediction consists in circumventing the lack of a large gold standard set from which models can be trained. Here we present a strategy based on positional bias and conservation, in order to improve signal detection. We were able to select word motifs with a common sequence conservation distribution profile regardless of their frequency of occurrence along introns. This approach resembles other motif discovery algorithms, such as PEAKS [37], which are also based on positional bias. However, the use of conserved instances alone allows for the production of differentiated distribution profiles, even for words occurring at very low frequency. Using BP-associated pentamers we built a set of high-confidence BP candidates by selecting functionally conserved instances. Remarkably, the abundance of the different motif variants correlates with U2 snRNA binding stability. There are, however, some exceptions, such as the CTCAN-containing nonamers that, despite their low binding stability, are quite frequent and highly conserved motif variants (see e.g. CTCAC pentamer in Figure 3 in Text S1). Nevertheless, the observed correlation, besides being a good indicator of the set quality, also suggests that the probabilistic score resulting from the statistical modeling of the signal might be representative of the signal strength, and therefore related to splicing efficiency.

### A predictive BP model

To build a BP predictive model, at least three main issues had to be tackled and improved over previously published methods. First, an adequate signal modeling: some positions in the signal might play a more prominent role in U2 binding stability compared to others, something not taken into account by Hamming distance-based scoring methods, and which appears to be partially responsible for their lower performance compared to methods based on a probabilistic description of the signal. Additionally, it was possible to detect subtle dependencies between adjacent positions within the signal, which we have exploited in a position-dependent 1^st^ order Markov model, yielding additional discriminative power over the use of a simple PWM. Moreover, the BP signal may be affected by the sequence biases of the intronic context. Indeed, the consensus BP motif in the training set differs between GC-rich and GC-poor introns. However, our model can recapitulate these properties, i.e. we predict the same consensus as expected for GC-rich introns and, likewise, for GC-poor introns (see Figure 16 in Text S1).

A second issue in the prediction of the BP is the association with other signals. The low IC of this signal in mammals suggests that BP selection may depend on additional signals in the pre-mRNA sequence. Accordingly, we considered features of the downstream PPT and incorporated them in the model using a machine-learning algorithm. Our benchmarking analysis demonstrates that additional PPT information improves accuracy over the probabilistic modeling of the signal alone, and over previously published methods. This improvement is particularly clear for cases in which the actual BP sequence is not a frequent motif variant. In fact, our results suggest that PPT information accounts for the majority of the accuracy difference between Gooding's and our SVM model. This reinforces the importance of modeling the relation between these two signals, as stronger PPTs might compensate for weaker BPs, but the lack thereof might even impair strong BP candidate recognition.

Finally, a third issue in BP prediction is the localization – even though BPs generally localize towards the region from 20 to 40 nucleotides upstream the 3SS, we show that BP localization is more dynamic than normally assumed. It appears to be highly dependent on the presence of AG dinucleotides in the 3′ end of the intron. In this work, and like in Gooding method [22], we search BPs in the AGEZ only. This contrasts with the other two methods (Plass [33] and Schwartz [32]) that, for every intron, scan a fixed region of 100 nts and 200 nts respectively, preferentially selecting hits that are closer to the 3SS. This fundamental difference might account for their lower prediction accuracy not only in the test set of mapped dBPs, but also in the set of proximal BPs, where searching over an unnecessarily long region can lead to the appearance of false positives. This may be particularly relevant for *ab initio* gene predictors that use BP information in their acceptor site models (see for instance [38]–[40]) by scanning over a fixed window size and do not consider more distant BPs as a possible configuration, which could lead to mispredictions.

### BP mapping in long AGEZ-containing introns

In this work we devoted special attention to the specific case of long AGEZ-containing introns. These are not only interesting from a computational point of view, as they represent an atypical kind of acceptor arrangement and potentially harbor a high number of BP candidates, but also from a biological perspective due to their association with regulated alternative splicing events. We experimentally mapped the BP for 5 introns characterized by the presence of long AGEZ in the 5′ terminus from *MBNL1*, *CLK1* and *CLK3* genes. One of the most striking and intriguing observations, for these introns and for the additional set of 4 introns from the *HTR4* gene, is the fact that more than one BP can be used. Remarkably, BPs often appear as doublets, with the BP adenosines in closely spaced positions, probably in association with the same PPT (see Figures 6, 7, 8, and 9 in Text S1). For *MBNL1* exon 9, the presence of a second population of lariats occurring at the later time point (180 minutes) of the *in vitro* splicing reaction also reflects the additional usage of two closely spaced BPs located at canonical positions. Interestingly, the splicing kinetics for this second population, which is associated to very weak BPs, appears to be much slower compared with the one in which the dBP is selected. Regardless of any mechanistic interpretation, the evidence presented here strongly supports that BP recognition in human introns can be more plastic than previously assumed, which probably ensures a greater resistance to BP disruptive mutations and/or allows for greater control over specific alternative splicing events. This hypothesis is in agreement with the observations in [34], where splicing of *HTR4* exons 3 and 5 is very resilient to mutations of the mapped BPs, being only impaired upon mutation of every surrounding adenosine, suggesting the use of additional cryptic BPs. Indeed it agrees with some of the earliest observations upon the effects of mutations upon mammalian BPs (reviewed in [41]).

In this set of experimentally validated introns, our SVM classifier had a modest performance compared to the previous benchmark. This is mainly explained by the fact that the mapped BPs for these introns are significantly different from the U2 complementary sequence (TACTAACAC). Additionally, for *CLK* introns the prediction is further complicated by the fact that PPTs downstream of the mapped BPs contain many purine interruptions. However, with the exception of *MBNL1* exon 9, in which the mapped dBP already ranked first according to the MM1, for all *MBNL1* and CLK mapped BPs, it is possible to observe a raise in prediction ranking from pure motif score (MM1) to SVM score. This adds extra evidence suggesting the importance of the PPT in BP recognition, as its associated features account for all the difference between MM1 and the final SVM model. Finally, considering the limitations of our training and the large numbers of candidates in long AGEZs, our results show that the SVM classifier is capable of delivering a good set of predictions for introns with long AGEZs.

### Human BPs

In order to refine our understanding on the relation between the BP and the AGEZ, we extraordinarily extended our search region to the last 500 nts of each intron. For approximately 5% of the introns, no candidate was found in the AGEZ, and in a fraction of those (0.44% of our human intron dataset) no TNA-containing 9-mers were found over the search region. These cases indicate the presence of a BP signal without the canonical TNA that, like for U12 type intron signals, will require independent modeling. On the remaining introns most of the best hits are located within the AGEZ towards the 5′ end of it. Interestingly, this distribution extends significantly up to 7/8 nucleotides upstream the AGEZ-defining AG-dinucleotide (*a3* in Figure 1). Beyond this distance, a previous study in yeast has shown that the BP proximal AG can, though at a low rate, be chosen, therefore affecting the recognition of the distal 3SS [42]. The distribution profile shown in Figure 6A strongly suggests that the region *r3* from Figure 1 might be shorter than it was previously assumed (12nts) and at the same time supports the initial assumption that the BP should be searched exclusively in the AGEZ.

Even though inter-AG dinucleotide distance appears to be determinant for the packed arrangement of BP, PPT and 3SS at the end of introns, our results suggest that large BP-3SS distances (within the AGEZ) might be related with a decrease in splicing efficiency, reflected by the higher prevalence of exon skipping and lower inclusion levels observed for exons preceded by more distant BPs. In these cases, stronger BP sequences and longer PPTs do not appear to have any compensatory effect in acceptor site recognition. Additionally, it has been demonstrated that distant BPs provide the opportunity for regulated alternative splicing through the binding of repressive regulatory factors in the extended region between the BP and the 3SS [23], [24], [43], [44], which could serve as further explanation to why such exons are more frequently skipped. Considering that long AGEZs are indicative of distant BPs, it is interesting to observe (see Figure 17 in Text S1) that their number has been increasing throughout the mammalian lineage at a similar rate for almost all the branches considered. This suggests, not only that newly formed distant BPs might provide an opportunity for new regulated alternative splicing events, but also that this process might be of evolutionary relevance in mammals. Another striking observation from our data is the inverse relation between BP sequence score and exon skipping, suggesting that BP-U2 binding stability might be of considerable importance for the overall splicing efficiency. Related to this, we have found that long introns tend to have weaker BP signals, whereas small introns show the opposite behaviour with the BP signal appearing more clearly defined, i.e. they contain fewer putative candidates and these have a stronger motif. This might be intrinsically associated to differences in the contribution of the different pre-mRNA signals responsible for exon definition, which is considered to be the prevailing mechanism of spliceosome assembly in mammals. With introns accounting for the majority of the primary transcript length, exons are early recognized through exon-spanning interactions between factors and corresponding signals, resulting in the combined recognition of the 5′SS and the upstream 3′SS [45]. In the context of long introns, sequence features other than the BP might be playing a more prominent role in exon recognition, which can potentially alleviate some of the contribution of the BP to early spliceosome assembly and splice site recognition in these cases.

Finally, previous studies have shown that AS is associated with exon creation [18], [35], [36]. It has been proposed that new exons are born with reduced splicing efficiency due to weaker splice sites and smaller differences between exonic and adjacent intronic content of splicing regulatory elements [18]. Here we explored the possibility that BP features might also be contributing to the low inclusion observed in recently created exons. In effect, our results suggest that the high rate of skipping observed for primate specific exons compared to mammalian ones results from a combination of poorly defined signals in the pre-mRNA, including the BP. Moreover, the weaker BP signals found in pseudo exons, with no inclusion evidence, underline the importance of the BP signal for accurate intron excision.

Splicing is a remarkably complex mechanism. The final configuration of mature mRNAs depends on an elaborate crosstalk between splicing factors and a myriad of potentially competing signals in the pre-mRNA molecule. The accurate identification of splicing signals, specially those that directly participate in the splicing reaction, may prove useful in the context of large scale analyses focusing on the characterization of disease-associated genomic mutations, as many might be directly related with alterations in the normal splicing patterns. In this paper we present a new and more accurate method for BP prediction in mammalian introns and provide new insights on acceptor site architecture. Our data strongly suggest that the BP conceals information relevant for acceptor site recognition and, therefore, it should be integrated in future splicing models.

## Methods

### Intron datasets

The genome sequences for 7 mammalian species (*Homo sapiens* – hg18; *Pan troglodytes* – PanTro2; *Macaca mulatta* – RheMac2; *Mus musculus* – mm9; *Rattus norvegicus* –RN4; *Canis familiaris* – CanFam2; and *Bos taurus* – BosTau4) and Refseq annotations for *Homo sapiens* were retrieved from the UCSC Genome Browser Database [46]. All introns preceding an internal exon and containing canonical splice sites were extracted from the annotation. After duplicate removal, there were 183187 unique introns in the human intron dataset. To obtain the corresponding orthologous introns in the other 6 species, the LiftOver tool [47] was used. Removing hit pairs that did not contain canonical splice sites or for which the flanks were in different strands and/or chromosomes, we obtained a set of 128790 orthologous introns in all 7 mammalian species. Additionally, we used three sets of introns preceding pseudo exons, primate specific exons and mammalian conserved exons, obtained from [18]. Pseudo exons were defined as sequence stretches of length comparable to real exons, intronic, located between apparently viable splice sites, not containing any termination codon in frame, and for which there is no evidence of inclusion. Additionally, if included in the mature transcript, they would not alter the reading frame.

### Positionally biased conserved pentamers

Out of the set of mammalian orthologous introns, the last 300 nt were aligned between all species using PRANK_+F_ [48] with default parameters. Only introns of length greater or equal to 300 nt in all species were considered (N = 98996). By scanning the alignments we were able to retrieve all pentamer instances that were exactly conserved in the 7 mammalian species, which we refer to as conserved instances. Their positions in the human sequence were recorded. Next we proceeded to the identification of pentamers that had a distribution of their conserved instances similar to the one expected for BPs, imposing the presence of an A preceded by a T 2 bases upstream: TNANN, NTNAN and NNTNA, which account for 184 unique pentamers. Thus, we selected pentamers according to three tests:

1. the distribution of their conserved instances over the last 300nt is non uniform, using a Kolmogorov-Smirnov test against a uniform distribution model (p-value<0.001).
2. the proportion of their conserved instances between positions −55nt and −15nt relative to the 3′ splice site is greater than expected, using χ^2^ test (p-value<0.001).
3. the conserved instances distribution peak lies between −55nt and −15nt relative to the 3′ splice site.

Using the above criteria, we were able to separate the initial set of 184 pentamers into three distinct groups based on their positional bias or lack of it: BP-associated, PPT-associated, and no association with any positionally biased signal. We first applied the two statistical tests 1) and 2) together. We discarded 37 pentamers that did not pass both, i.e. they do not show any positional bias in occurrence or conservation. We applied both tests simultaneously since some pentamers, like TCACG, TTACG or TAACG, would pass test 2) but their total count is very low and their occurrence in the range −55nt to −15nt might be just due to chance. These cases with low counts got discarded because they failed the test 1). Out of the remaining 147, 23 had a peak outside the region of interest. Visual inspection of these 23 shows that they're Py-rich and their bias region lies between −15 and 0, thus we label them as PPT-associated. We were thus left with 124 pentamers that do not present a uniform distribution of conservation in the last 300 bp of the intron, their occurrences are more frequent than expected in the region between −55 to −15, and their distribution peak is inside that same region. These were considered BP-associated.

### Reference set of conserved putative BPs

In order to build a set of putative branch points imposing minimal sequence bias, we devised a 2 step strategy based on positional bias and conservation. First, we identified all introns for which there is only one TNA conserved in all 7 species, as they will more likely include a BP candidate. Moreover, each unique instance must be positioned between 15 and 55 nts upstream the 3SS. For every instance, the human nonamer containing the TNA motif in the central position was collected – *consTNA* set. Second, from those instances, we only kept the ones overlapped by at least one of the previously determined BP-associated pentamers in every one of the species considered – *consTNA-BP5* set.

### U2 binding energy

For every possible nonamer, containing a T and an A in positions 4 and 6, respectively, we used the program RNAcofold from the Vienna RNA package [49] to calculate its binding energy to the U2 snRNA. We forced the complete pairing of all nucleotides between the two sequences, with the exception of the BP adenosine, which was forced not to pair with any nucleotide from the U2 snRNA sequence. The energy of the base pairing depends on the complementarity between both sequences, the length of the sequence, and the sequence composition. If the energy is high (negative but close to zero), the base pairing is very unstable, because the complementarity of the sequences is poor. Conversely, if the energy is very negative, the base pairing is much more stable. Nonamers containing the same core region (5 central nucleotides) were grouped together and mean energy was computed for each cluster.

### Information content

For every column 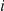 in the *consTNA-BP5* set, the information content (*IC*) a measure of conservation was computed according to the formula:
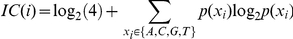
where 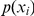 is the probability of finding the nucleotide 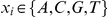 in position 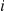


### Mutual information

In order to test possible association between different BP positions we computed the Mutual Information (MI), using all human putative BPs in the *consTNA-BP5* set, between all possible 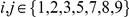 position pairs according to the formula:
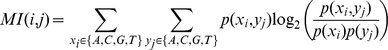
where 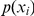 is the probability of finding the nucleotide 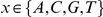 in position 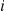, 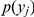 is the probability of finding the nucleotide 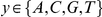 in position 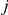, and 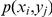 is the joint probability of simultaneously finding a particular combination of nucleotides 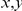 in positions 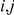, respectively.

### Branch point sequence score

According to the 1^st^ order dependencies detected by MI, we modeled the BP signal making use of a position-dependent Markov model. Due to the fact that positions 4 and 6 are fixed as T and A respectively, to compute the conditional probabilities of their downstream positions, 2^nd^ order dependencies were considered. Accordingly, the probability of occurrence of the nonamer 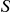 in a model, omitting positions 4 and 6, can be represented as:
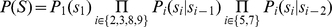
where 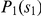 is the probability of finding the nucleotide 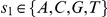 in the first position (considered independent) of the model and 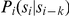 is the conditional probability of finding the nucleotide 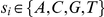 in position 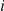 of the model assuming nucleotide 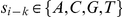, in position 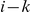. This was computed for every BP candidate, taking as reference a positive set (*consTNA-BP5* set) and a negative set composed of randomly selected intronic nonamers that contained T and A in positions 4 and 6 respectively. The final motif score 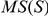 is given by the formula:
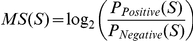
and it reflects how likely a given sequence belongs to the positive set relative the negative set.

### Polypyrimidine tract prediction

A heuristic method was used in order to identify potential polypyrimidine tracts. Sequences were scanned by a Python program that finds all subsequences with the following characteristics, maximizing for length:

1. Both 3′ and 5′ ends must be pyrimidines;
2. No more than two contiguous purines are allowed;
3. Every purine segment (length L<3) must be surrounded by at least 4L pyrimidines (this forces a minimum pyrimidine content greater than 2/3) distributed in a way that both upstream and downstream pyrimidine segments are of length greater or equal to L;
4. T(GT)_n_ stretches are allowed;
5. Minimum length of 9nt or uridine content greater or equal to 5.

For every predicted PPT a score was calculated based on the sequence length and content according to the following formula:
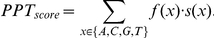
Where 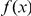 is the absolute frequency of nucleotide 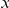 in the PPT and 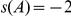, 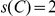, 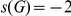 and 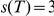. This scoring scheme has been previously used in [50].

### Branch point prediction

Due to the low information content, BP prediction cannot simply rely on the statistical modeling/scoring of the signal. In fact, there are other additional factors responsible for the recognition of the BP in mammalian introns. Accordingly, to build a predictive BP model, the sequence score was combined with PPT associated features using a Support Vector Machine (SVM) algorithm. The aim was to score the candidates based on the SVM score which is the distance in feature space between the candidate and the decision boundary. The *consTNA-BP5* set was used as positive set for training. As negative set we picked all other nonamers, in this intron set, containing TNA in the central positions. For every candidate, 4 features were collected:

1. Sequence score using the order 1 Markov model;
2. Pyrimidine content between the putative BS and the 3SS;
3. Distance to the closest downstream PPT.
4. Score of the closest downstream PPT.

These features and the classification (as positive or negative) served as input to SVM^LIGHT^ [51]. For balanced learning, an equal number of positive and negative cases were used. The resulting predictive model was used to systematically score BP candidates. BP predictions for 183187 human introns can be found in http://regulatorygenomics.upf.edu/SVM_BP/BP_predictions.tar.gz.

Additionally, we have developed a web-tool where the algorithm can be run for multiple intronic sequences. The web-tool and a stand-alone version of the software are available at the URL http://regulatorygenomics.upf.edu/SVM_BP/.

### *In vitro* splice constructs

The BP and the PY-tract of the PY7 reporter containing exon 2 and 3 of α-tropomyosin [52] were replaced with individual AGEZs by cloning them using following primers via XhoI and PvuII or AluI (restriction sites are underlined):

*MBNL1* Exon 6 forward 5′-GTGCTCGAGCCAATAACAACTCAGTAGTGCC;

*MBNL1* Exon 6 reverse 5′-TTATTAGCTTAATTAGCAGGCAGCGAGCAC;

*MBNL1* Exon 8 forward 5′- GTGCTCGAGGGCTTTTATTCTTCACTTGAGAC;

*MBNL1* Exon 8 reverse 5′- TTATTCAGCTGCCCATCATGCATTGCAAC;

*MBNL1* Exon 9 forward 5′- GTGCTCGAGTTTTTGACTTAGCATATTAAGCCTG;

*MBNL1* Exon 9 reverse 5′- CTTTCGGAGGGAAAATCATATAAGC (used for blunt end cloning to preserve suboptimal 3′ splice site);

*CLK1* Exon 4 forward 5′- GTGCTCGAGTTCAGTGAATGCTACAACTAAGC;

*CLK1* Exon 4 reverse 5′-TTATTCAGCTGGAAACGTCAAGTGGGCG

*CLK3* Exon 4 forward 5′- GTGCTCGAGGTTTTCTTTACATACCTGTAGCTG

*CLK3* Exon 4 reverse 5′- TTATTCAGCTGCATGCACCGCCCCCC

### *In vitro* splicing and primer extensions

Py7 constructs were linearized with XbaI prior to *in vitro* transcription with SP6 polymerase. *In vitro* transcription and splicing were carried out as previously described [23], [53]–[55]. 100 fmol of ^32^P-5′-labelled primer were hybridized to 100 fmol of spliced, debranched or control RNA template at the most 3′end of the intron and annealing was allowed for 30 minutes at 42°C. Lariat branch points were mapped by extending with 10 units of AMV reverse transcriptase (Promega) for 45 minutes at 42°C and by comparing the resulting terminations in the RT to the ones of debranched and control RNA. Primer extension reactions were loaded on 8% denaturing polyacrylamide gels side by side with sequencing reactions with the same primers and appropriate plasmid templates using T7 DNA polymerase.

### EST inclusion levels

EST alignments were retrieved from UCSC Genome Browser Database [46] and compared with the annotations. For each exon, the percentage of EST inclusion level is defined as
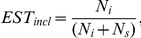
where 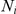 is the number of ESTs including the exon and 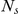 the number of ESTs that cover the genomic region of the exon but skip it. This measure was calculated for all the exons preceded by introns in the human intron dataset. Only exons with 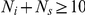 were considered, accounting for a total of 78186. Some exons have zero EST inclusion, as all the corresponding ESTs show exon skipping, but their existence is supported by mRNA evidence.

## Supporting Information

Dataset S1 Training and benchmarking datasets. It provides coordinates in hg18 of the introns and BPs used for training and testing.
(0.37 MB TAR)

Text S1 Supplemental Table 1 and Figures 1–17.
(6.30 MB PDF)
