# Supplementary material for: Genome-Wide Association between Branch Point Properties and Alternative Splicing
Source: PLoS Comput Biol. 2010 Nov 24;6(11):e1001016. doi: 10.1371/journal.pcbi.1001016 (PMC2991248; doi:10.1371/journal.pcbi.1001016)
Supplement: Text S1 — Supplemental Table 1 and Figures 1–17. (6.30 MB PDF) [file pcbi.1001016.s002.pdf]

Supplemental File 1

Title:
Genome-wide Association between Branch Point Properties and Alternative Splicing

Authors:
André Corvelo [1,4], Martina Hallegger [2], Christopher W. J. Smith [2] and Eduardo Eyras [1,3,§]

[1]Computational Genomics, Universitat Pompeu Fabra, Aiguader 88, Barcelona, 08003, Spain

[2]Department of Biochemistry, University of Cambridge, Tennis Court Road, Cambridge CB2 1QW, UK

[3]Catalan Institution for Research and Advanced Studies, Passeig Lluís Companys 23, Barcelona, 08010, Spain

[4]Graduate Program in Areas of Basic and Applied Biology, Universidade do Porto, Praça Gomes Teixeira, Porto, 4099-002, Portugal

[§]Corresponding author. eduardo.eyras@upf.edu

Table S1 –Highly abundant pentamers in the region between -15 and -55 nts relative to the 3SS in human introns. P values inferred by means of a Z-score.

p-value	Word	Counts (N)
p < 0.001	ttttt	60196
	atttt	33256
	tttct	32162
	ttttc	27494
	tattt	26478
	tgttt	26408
	ctttt	26375
	**tttta**	26149
	tcttt	25467
	ttctt	24890
p < 0.01	tctct	22500
	**tttaa**	22448
	tttgt	21530
	**tttat**	21422
	**ttatt**	21398
	gtttt	20731
	ttttg	20281
	aattt	19975
	aaaaa	19724
	ttgtt	19260
p < 0.05	aaaat	18902
	ccctg	18867
	**ttaaa**	18277
	cattt	18063
	ctctc	18042
	**taatt**	17824
	cctct	17743
	ttcct	17651
	ttctc	17585
	tgtgt	17578
	ccctc	17324
	ctctg	17256
	**taaaa**	17234
	**ttaat**	17062
	cctcc	16975
	ctccc	16673
	cttct	16561
	ttctg	16137
	ctcct	16080
	tccct	16015

TNA-containing pentamers in bold.

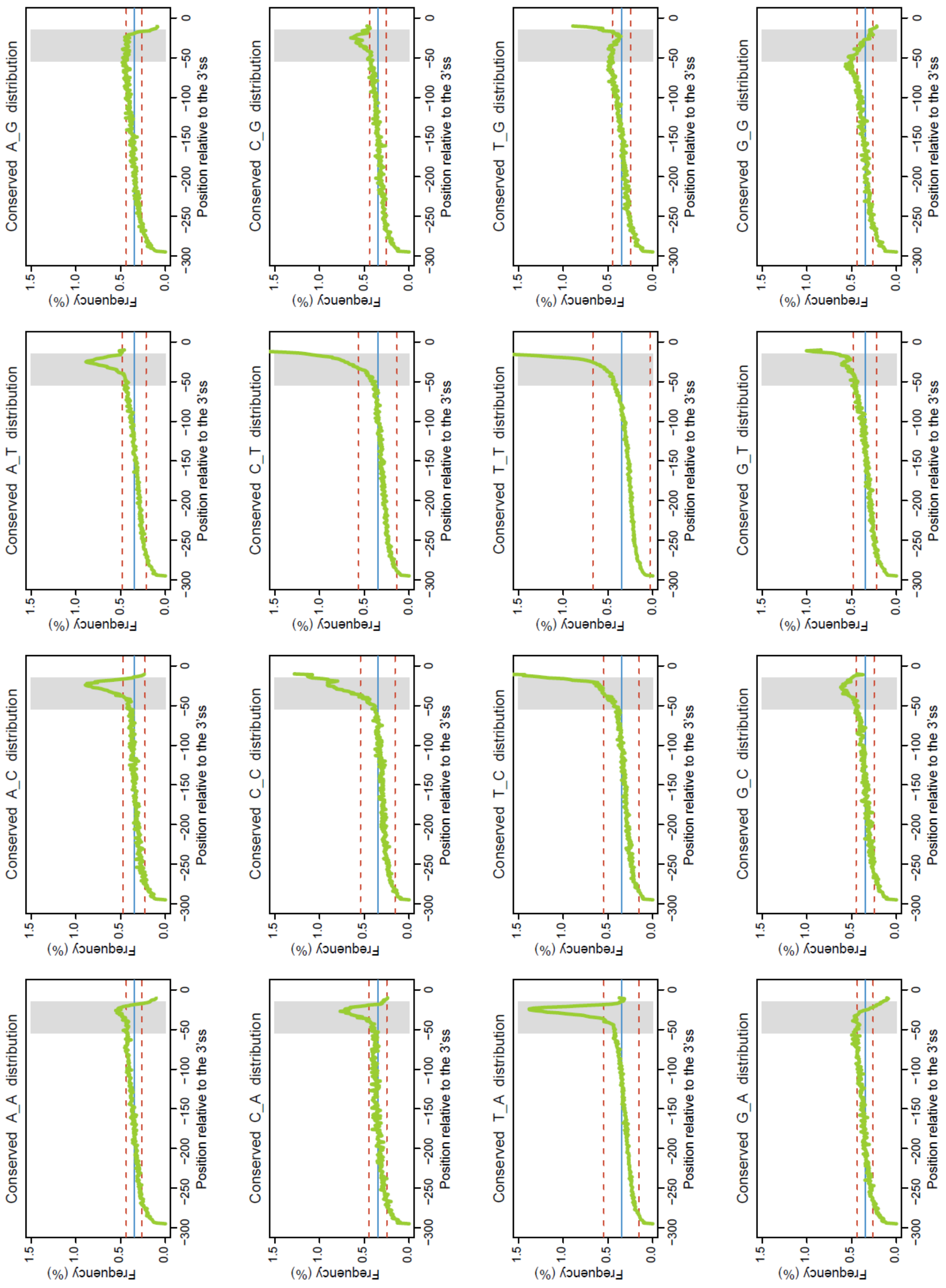

Figure S1 – Conserved N_N distribution biases.

Distribution of mammalian wide conserved N_N instances in the last 300nt of human introns. All combinations (16) are shown independently. The blue line represents the mean frequency. The dashed red lines represent the mean+- the standard deviation.

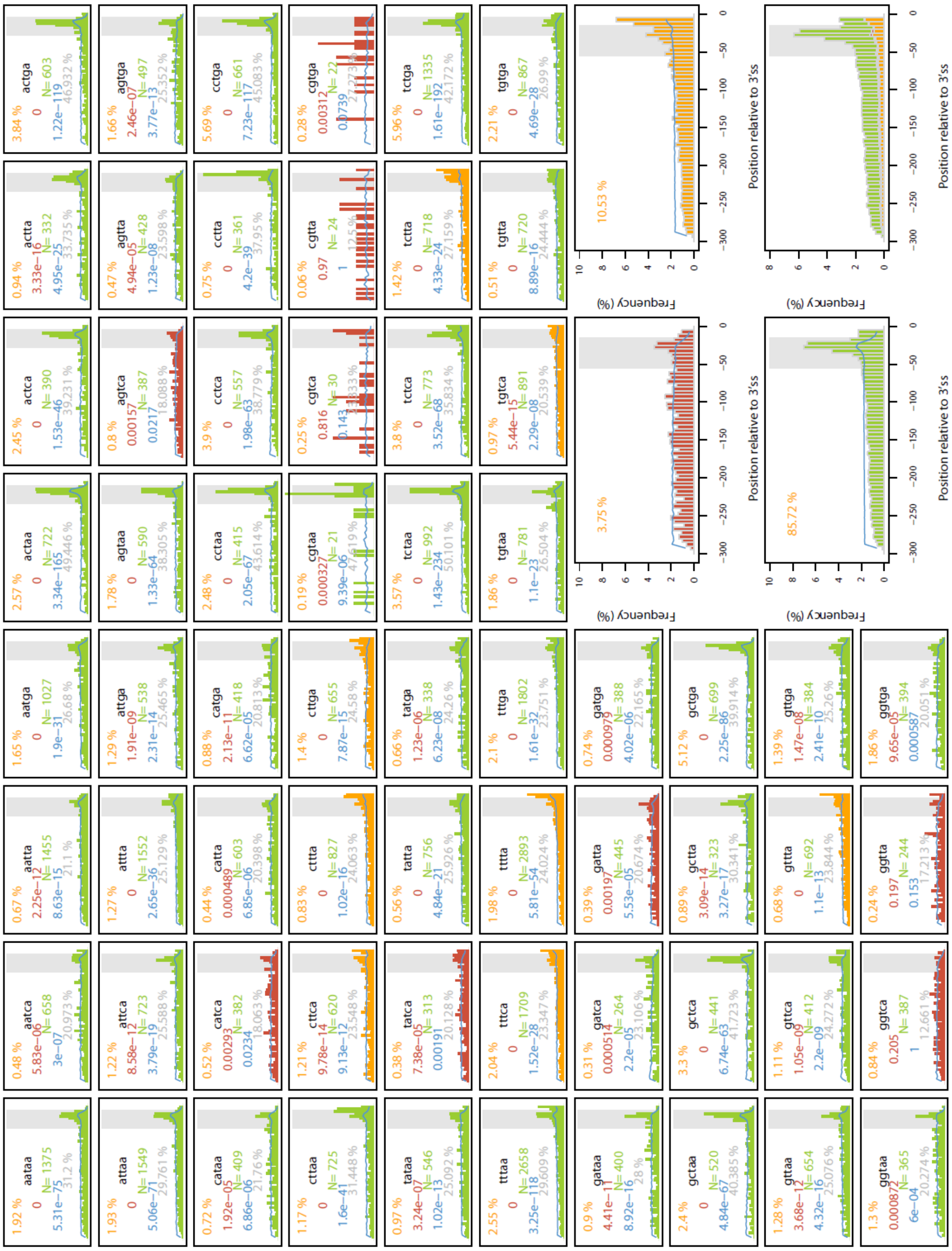

Figure S2 – Positional bias of conserved NNTNA pentamers in human introns.
The frequency of each pentamer was computed on 5nt bins over the last 300nts of human introns. Pentamers labeled as BP-associated are represented in green, PPT-associated in orange and with no association with any positionally biased signal in red (see Main Manuscript for details). The blue line represents frequency of all (conserved and non-conserved) instances. y-axis ranges from 0 to 20 % and the x-axis represents the region comprehended between -300 to 0 nts upstream the 3SS. The four plots in the bottom-right represent the distribution of all NNTNA pentamers together grouped by category. Text in the plots: black – pentamer sequence; green – number of conserved instances; grey – percentage of conserved instances in the region between 15 and 55 nts upstream de 3SS; orange – percentage of the *consTNA* set; red – KS test against a uniform distribution p-value; blue – Chi-square test for overrepresentation in the region between 15 to 55 nts upstream de 3SS p-value.

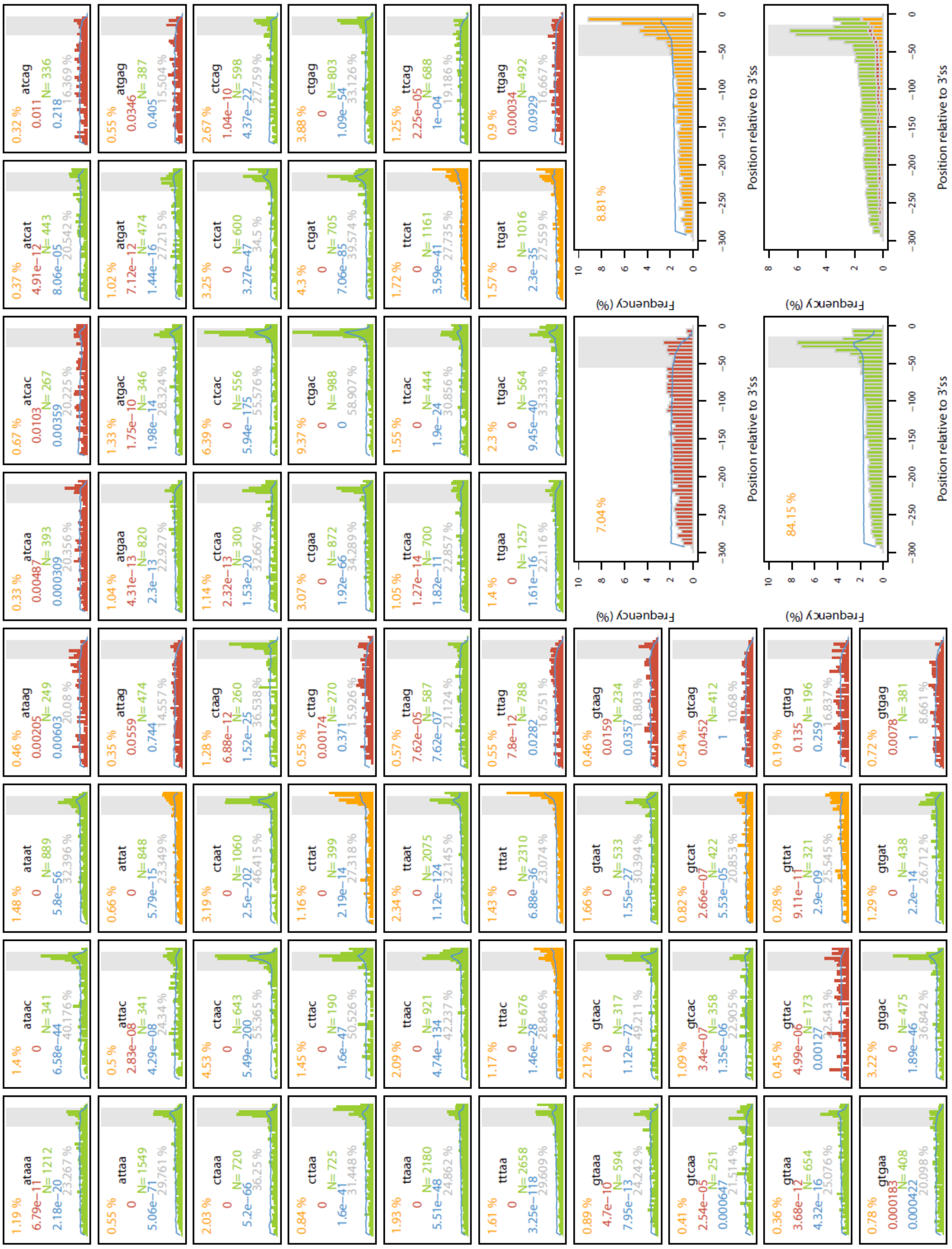

Figure S3 – Positional bias of conserved NTNAN pentamers in human introns.
The frequency of each pentamer was computed on 5nt bins over the last 300nts of human introns. Pentamers labeled as BP-associated are represented in green, PPT-associated in orange and with no association with any positionally biased signal in red (see Main Manuscript for details). The blue line represents frequency of all (conserved and non-conserved) instances. y-axis ranges from 0 to 20 % and the x-axis represents the region comprehended between -300 to 0 nts upstream the 3SS. The four plots in the bottom-right represent the distribution of all NTNAN pentamers together grouped by category. Text in the plots: black – pentamer sequence; green – number of conserved instances; grey – percentage of conserved instances in the region between 15 to 55 nts upstream de 3SS; orange – percentage of the *consTNA* set; red – KS test against a uniform distribution p-value; blue – Chi-square test for overrepresentation in the region between 15 to 55 nts upstream de 3SS p-value.

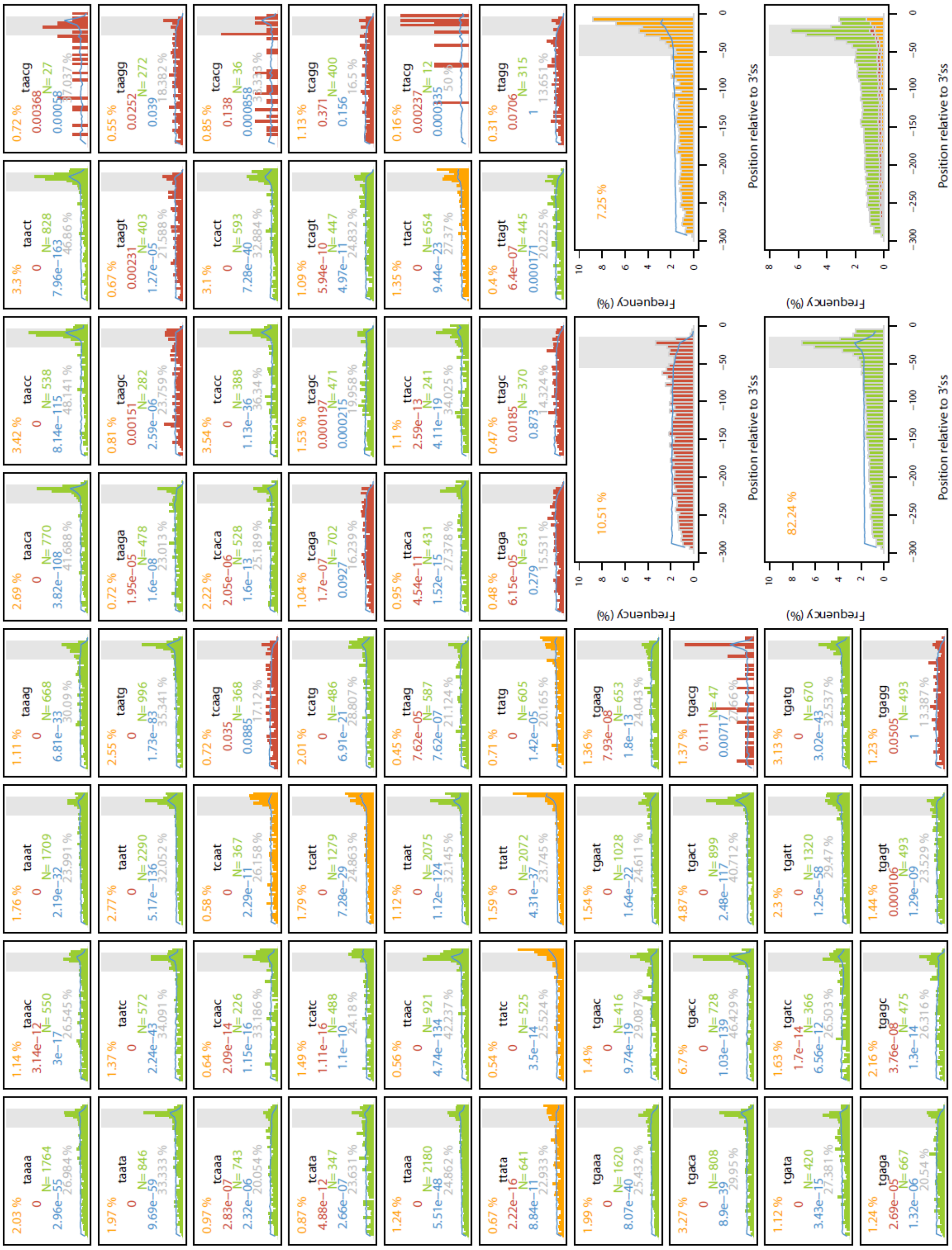

Figure S4 – Positional bias of conserved TNANN pentamers in human introns.
The frequency of each pentamer was computed on 5nt bins over the last 300nts of human introns. Pentamers labeled as BP-associated are represented in green, PPT-associated in orange and with no association with any positionally biased signal in red (see Main Manuscript for details). The blue line represents frequency of all (conserved and non-conserved) instances. y-axis ranges from 0 to 20 % and the x-axis represents the region comprehended between -300 to 0 nts upstream the 3SS. The four plots in the bottom-right represent the distribution of all TNANN pentamers together grouped by category. Text in the plots: black – pentamer sequence; green – number of conserved instances; grey – percentage of conserved instances in the region between 15 to 55 nts upstream de 3SS; orange – percentage of the *consTNA* set; red – KS test against a uniform distribution p-value; blue – Chi-square test for overrepresentation in the region between 15 to 55 nts upstream de 3SS p-value.

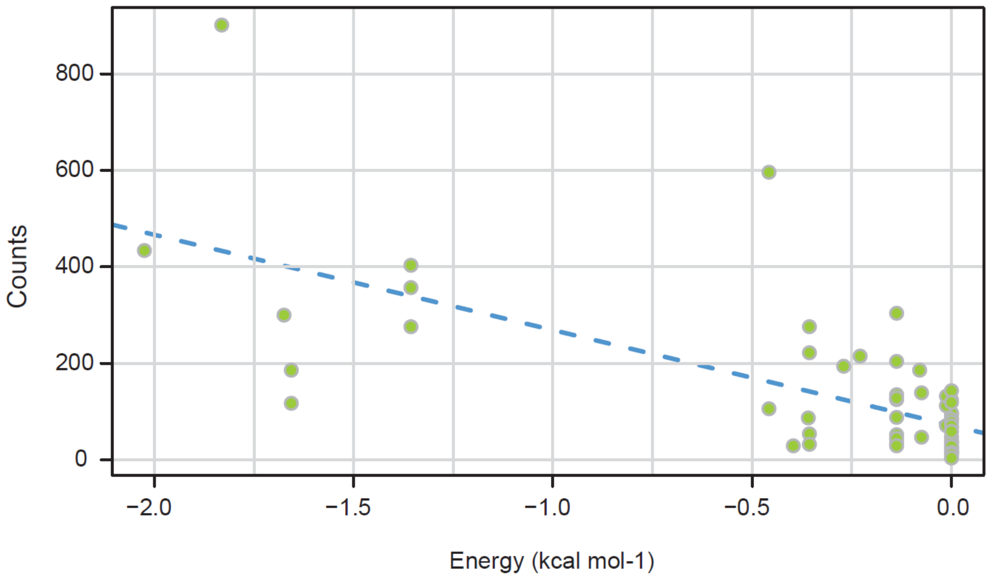

Figure S5 – Binding Energy versus motif abundance.
Scatter plot of BP-U2 binding energy versus counts for the all *consTNA-BP5* 9-mers, clustered by core pentamer (see manuscript for details).

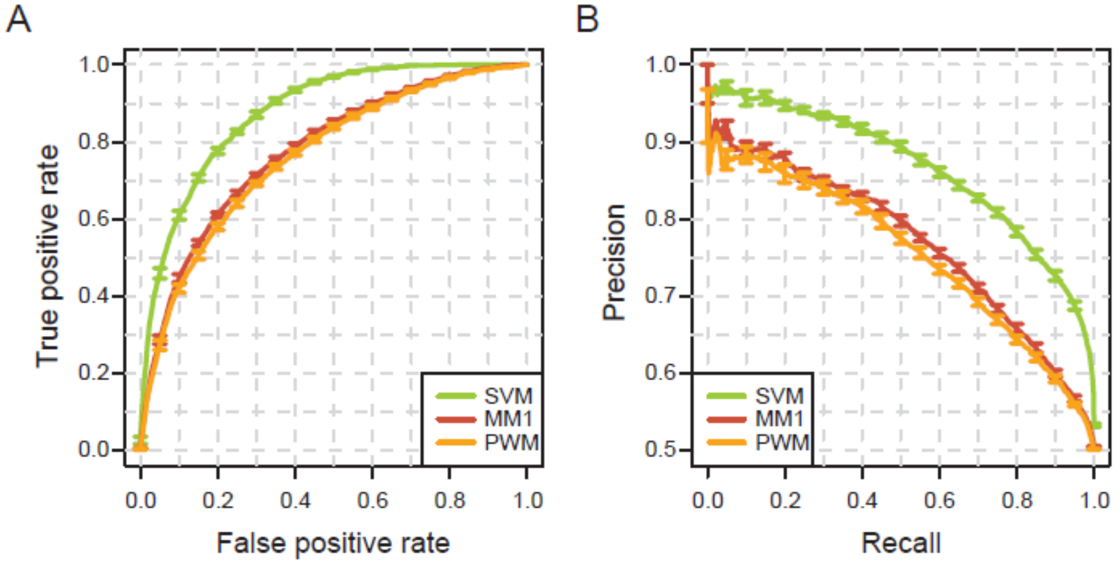

Figure S6 – ROC and Precision/recall curves for 3 classifiers, obtained on a 10-fold cross-validation.
A – ROC curve for the SVM (green), a order 1 Markov model (red) and a Position Weight Matrix (orange), obtained on a 10-fold cross-validation. **B** – Precision recall curves for the same 3 classifiers. The bars represent the standard error over 10 runs.

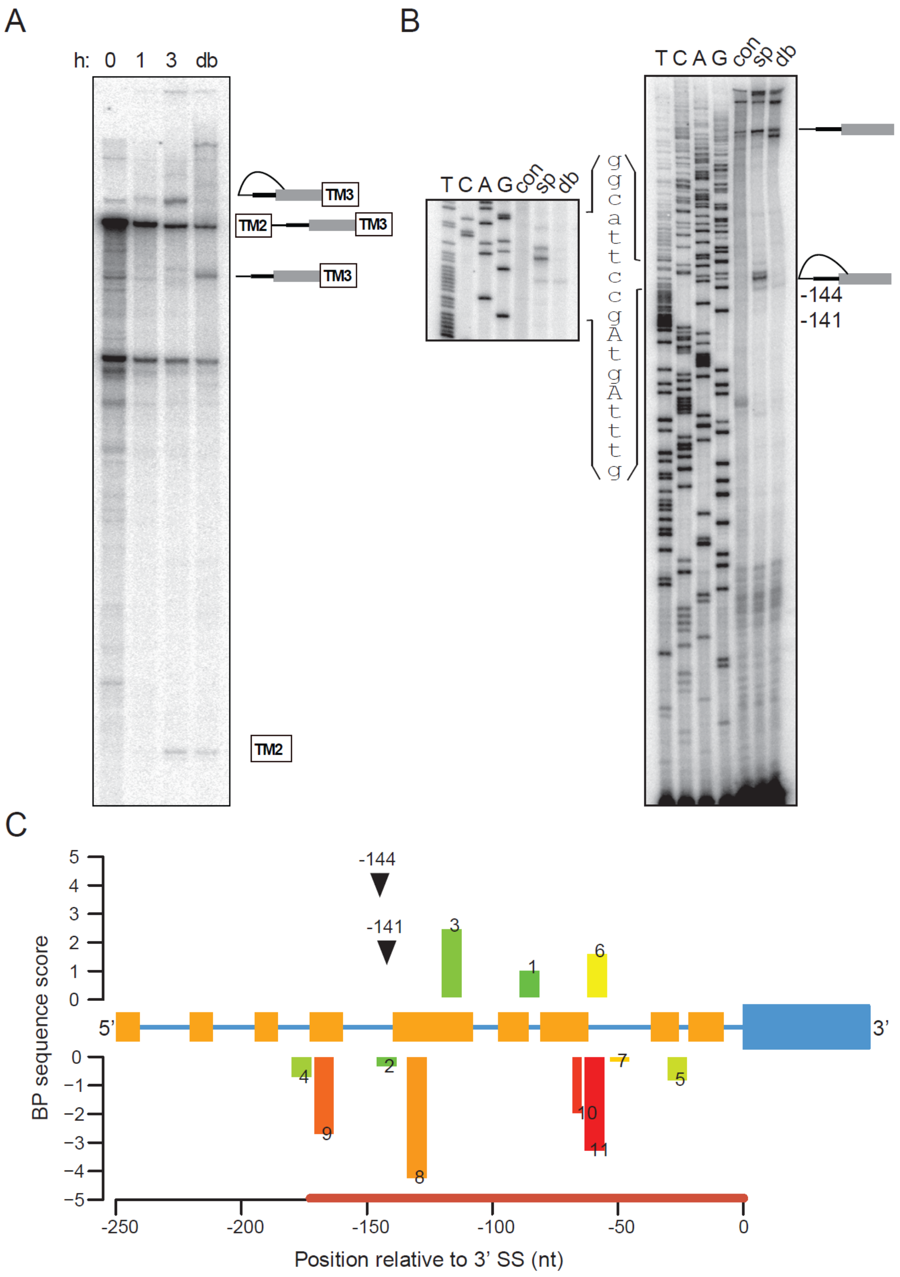

Figure S7 – Mapping BPs of *MBNL1* exon6.

A – In vitro splicing of the construct containing the AGEZ preceding *MBNL1* exon 6. Splicing reactions were incubated from 0 to 180 minutes. A sample from the 180 minute time point was subsequently debranched (lane db). Increasing amounts of splicing intermediates and products accumulate over the 180 minutes time course. **B** – Primer extensions map BPs to positions -141 and -144. Right panel shows primer extension and sequencing with primer hybridizing to the most 3' end of the intron. The sequences surrounding the BPs are depicted on the left side of the gel. Left panel shows a detail of primer extension and sequencing surrounding the both BPs. **C** – BP predictions on the AGEZ plus 12 upstream bases using the SVM classifier. Intronic and exonic regions are shown as a blue line and a box, respectively. Predicted PPTs, are shown as orange boxes over the intron. The AGEZ is represented as the red thick line over the x-axis. Each BP candidate is represented by a bar, in which its height represents the sequence score according to the MM1 model. Bars have been colored according to their SVM ranking in total set of predictions for the considered region, from green (1st) to red (last). The ranking is also annotated in the top of each bar. Black arrowheads point the location of the mapped BPs.

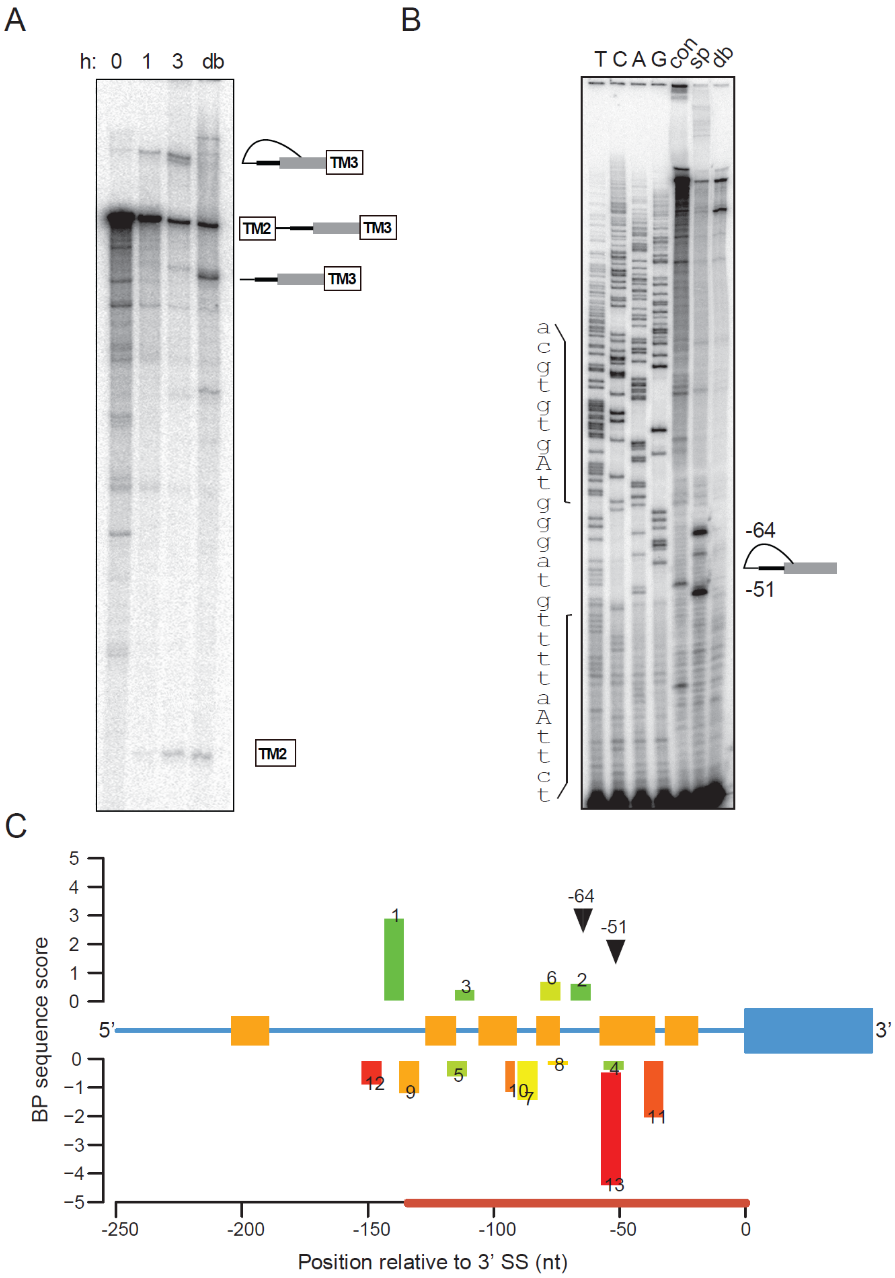

Figure S8 - Mapping BPs of *MBNL1* exon8.

A – In vitro splicing of the construct containing the AGEZ preceding *MBNL1* exon 8. Splicing reactions were incubated from 0 to 180 minutes. A sample from the 180 minute time point was subsequently debranched (lane db). Increasing amounts of splicing intermediates and products accumulate over the 180 minutes time course. **B** – Primer extensions map BPs to positions -51 and -64. Panel shows primer extension and sequencing with primer hybridizing to the most 3' end of the intron. The sequences surrounding the BPs are depicted on the left side of the gel. **C** – BP predictions on the AGEZ plus 12 upstream bases using the SVM classifier. Intronic and exonic regions are shown as a blue line and a box, respectively. Predicted PPTs, are shown as orange boxes over the intron. The AGEZ is represented as the red thick line over the x-axis. Each BP candidate is represented by a bar, in which its height represents the sequence score according to the MM1 model. Bars have been colored according to their SVM ranking in total set of predictions for the considered region, from green (1st) to red (last). The ranking is also annotated in the top of each bar. Black arrowheads point the location of the mapped BPs.

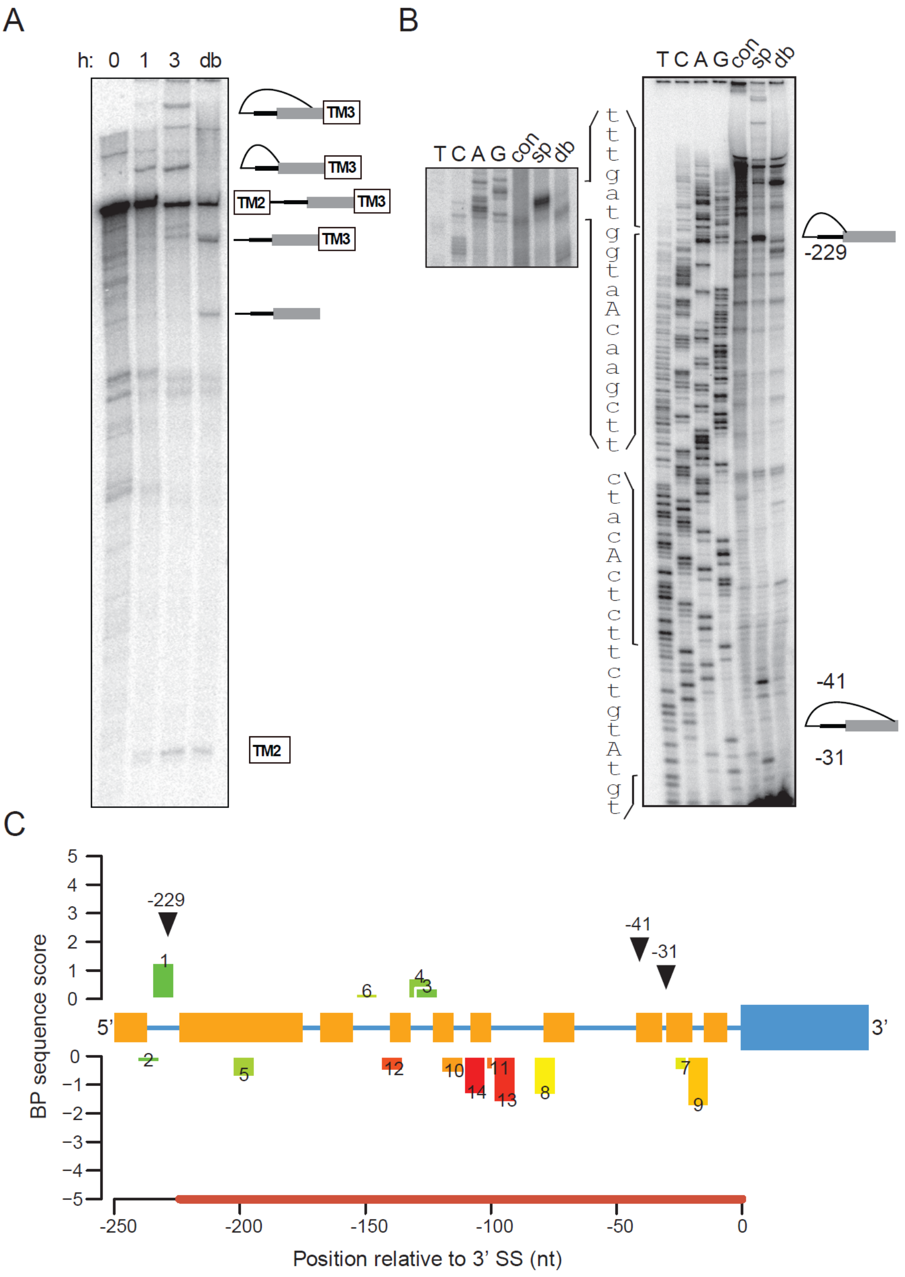

Figure S9 - Mapping BPs of *MBNL1* exon9.

A – In vitro splicing of the construct containing the AGEZ preceding *MBNL1* exon 9. Splicing reactions were incubated from 0 to 180 minutes. A sample from the 180 minute time point was subsequently debranched (lane db). Increasing amounts of splicing intermediates and products accumulate over the 180 minutes time course. The two different populations of lariats correspond to differently positioned BPS. **B** – Primer extensions map BPs to positions -31, -41, and -229. Right panel shows primer extension and sequencing with primer hybridizing to the most 3' end of the intron. The sequences surrounding the BPs are depicted on the left side of the gel. Left panel shows a detail of primer extension and sequencing surrounding the BP located at position -229. **C** – BP predictions on the AGEZ plus 12 upstream bases using the SVM classifier. Intronic and exonic regions are shown as a blue line and a box, respectively. Predicted PPTs, are shown as orange boxes over the intron. The AGEZ is represented as the red thick line over the x-axis. Each BP candidate is represented by a bar, in which its height represents the sequence score according to the MM1 model. Bars have been colored according to their SVM ranking in total set of predictions for the considered region, from green (1st) to red (last). The ranking is also annotated in the top of each bar. Black arrowheads point the location of the mapped BPs.

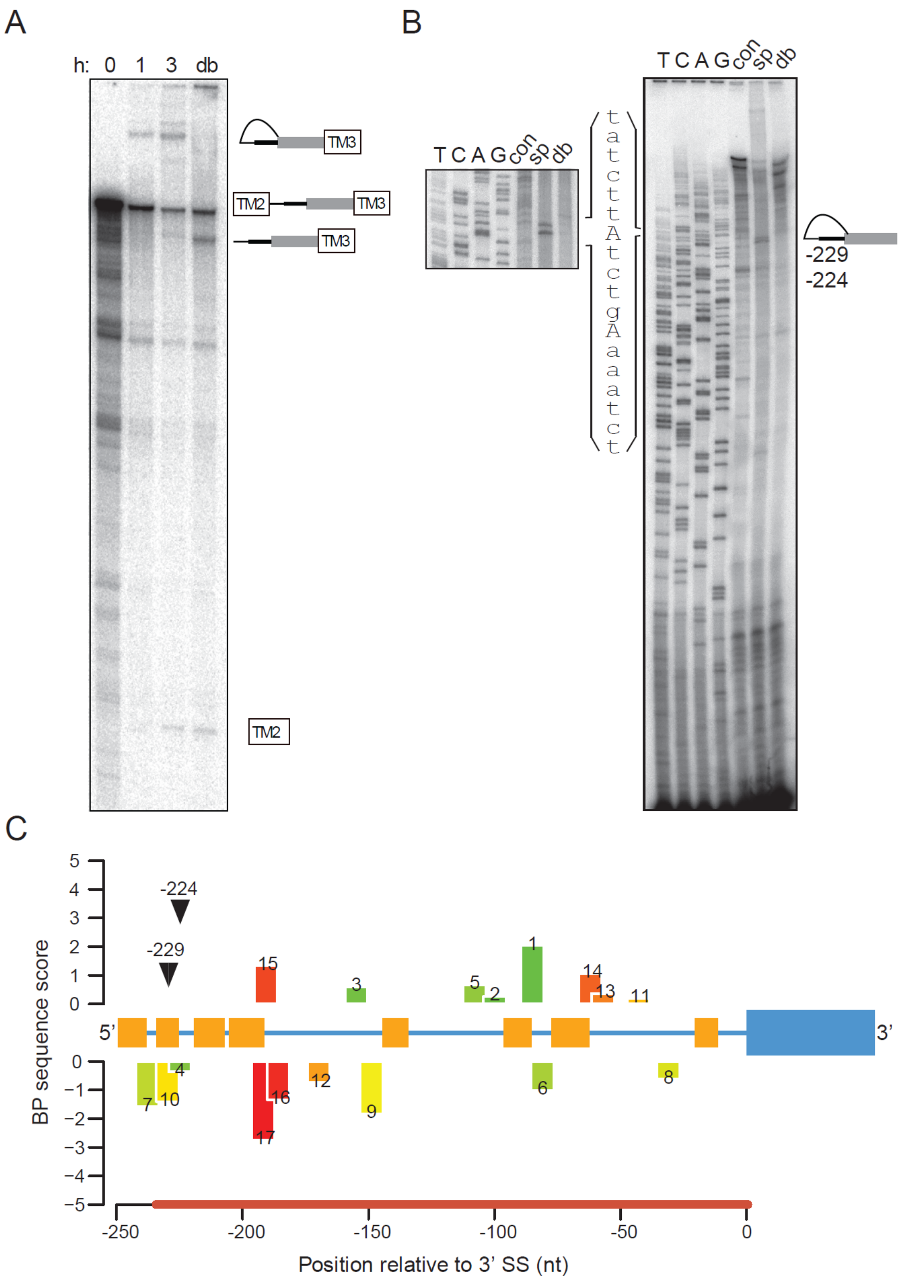

Figure S10 - Mapping BPs of *CLK1* exon4.

A – In vitro splicing of the construct containing the AGEZ preceding *CLK1* exon 4. Splicing reactions were incubated from 0 to 180 minutes. A sample from the 180 minute time point was subsequently debranched (lane db). Increasing amounts of splicing intermediates and products accumulate over the 180 minutes time course. **B** – Primer extensions map BPs to positions -224 and -229. Right panel shows primer extension and sequencing with primer hybridizing to the most 3' end of the intron. The sequences surrounding the BPs are depicted on the left side of the gel. Left panel shows a detail of primer extension and sequencing surrounding both BPs. **C** – BP predictions on the AGEZ plus 12 upstream bases using the SVM classifier. Intronic and exonic regions are shown as a blue line and a box, respectively. Predicted PPTs, are shown as orange boxes over the intron. The AGEZ is represented as the red thick line over the x-axis. Each BP candidate is represented by a bar, in which its height represents the sequence score according to the MM1 model. Bars have been colored according to their SVM ranking in total set of predictions for the considered region, from green (1st) to red (last). The ranking is also annotated in the top of each bar. Black arrowheads point the location of the mapped BPs.

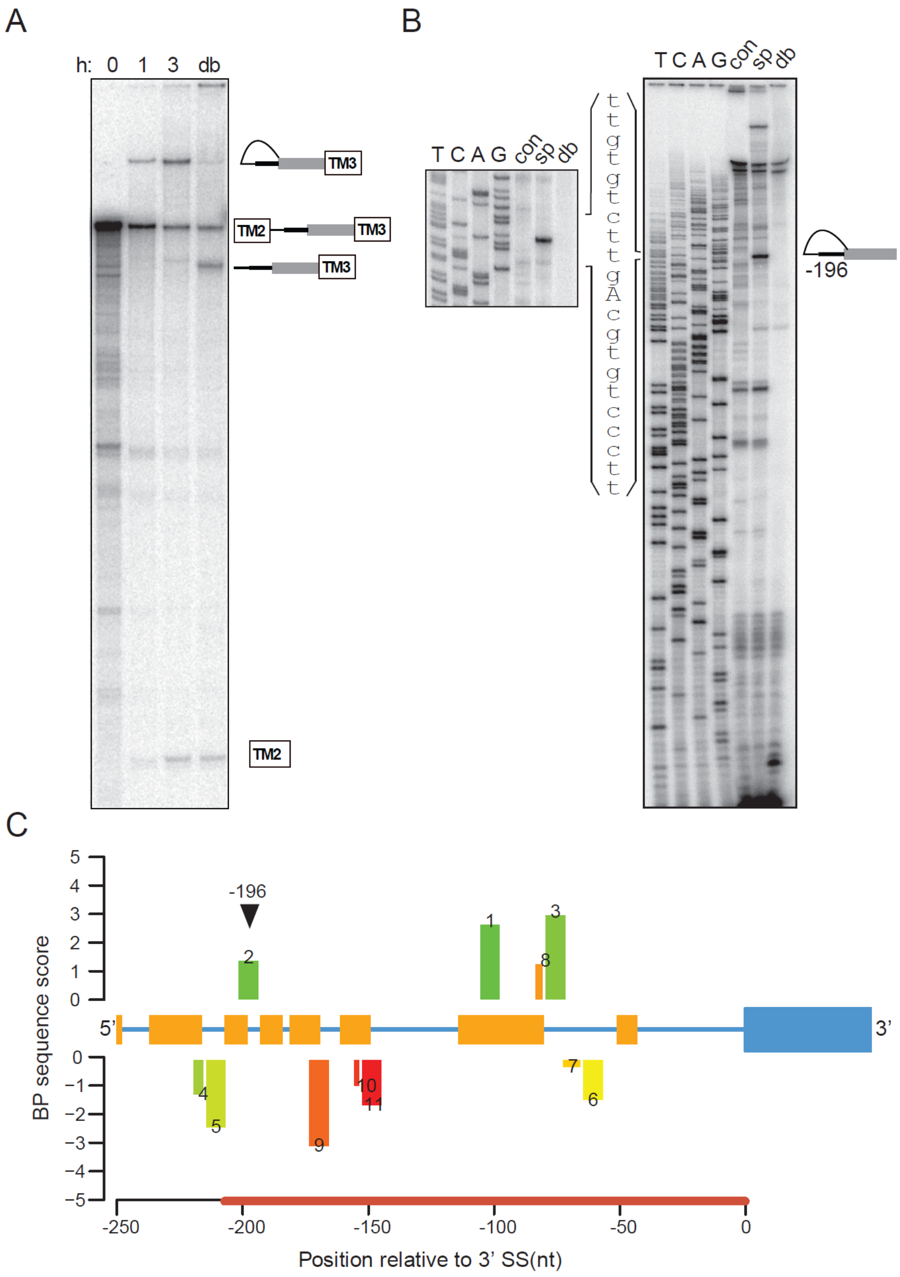

Figure S11 - Mapping BPs of *CLK3* exon4.

A – In vitro splicing of the construct containing the AGEZ preceding *CLK3* exon 4. Splicing reactions were incubated from 0 to 180 minutes. A sample from the 180 minute time point was subsequently debranched (lane db). Increasing amounts of splicing intermediates and products accumulate over the 180 minutes time course. **B** – Primer extensions map the BP to position -196. Right panel shows primer extension and sequencing with primer hybridizing to the most 3' end of the intron. The sequences surrounding the BP are depicted on the left side of the gel. Left panel shows a detail of primer extension and sequencing surrounding the BP located at position -196. **C** – BP predictions on the AGEZ plus 12 upstream bases using the SVM classifier. Intronic and exonic regions are shown as a blue line and a box, respectively. Predicted PPTs, are shown as orange boxes over the intron. The AGEZ is represented as the red thick line over the x-axis. Each BP candidate is represented by a bar, in which its height represents the sequence score according to the MM1 model. Bars have been colored according to their SVM ranking in total set of predictions for the considered region, from green (1st) to red (last). The ranking is also annotated in the top of each bar. Black arrowhead points the location of the mapped BP.

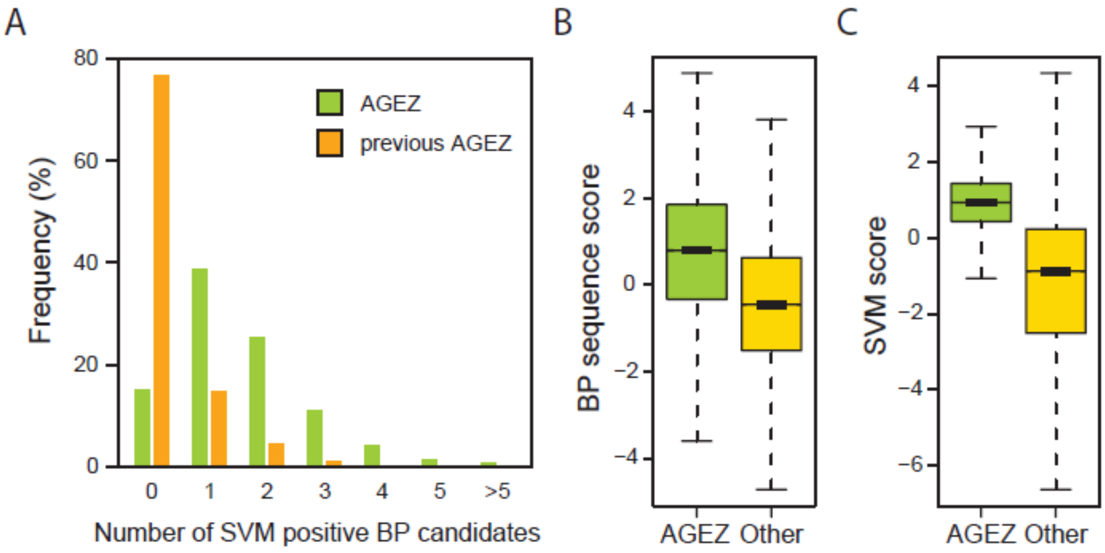

Figure S12 – The 1st AGEZ presents a higher number of positive scoring candidates.
A – Histogram showing the distribution of the number of positive BP candidates in the 1st AGEZ (green) and in the next upstream AGEZ (orange) AGEZs. Boxplots of sequence score (**B**) and SVM score (**C**) for BP candidates inside the 1st AGEZ compared to BP candidates outside this region.

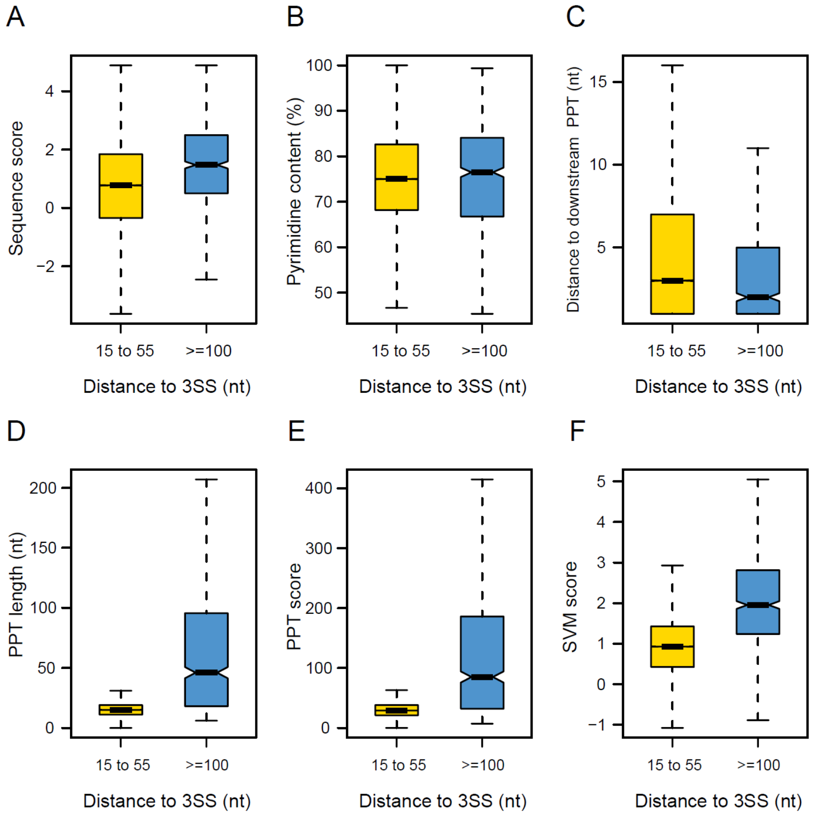

Figure S13. Comparison of the properties of distant and close BPs.
Boxplots for the properties of close BPs (yellow), located at distances 15 to 55 nt from the 3SS, and distant BPs (dBPs) (blue), defined as located beyond 100 nt from the 3SS. The properties tested are, from left to right and from top to bottom: BP sequence score (**A**), pyrimidine content between the BP and the 3SS (**B**), distance to the nearest PPT (**C**), length of the PPT (**D**), score of the PPT (**E**), SVM score for the BP (**F**).

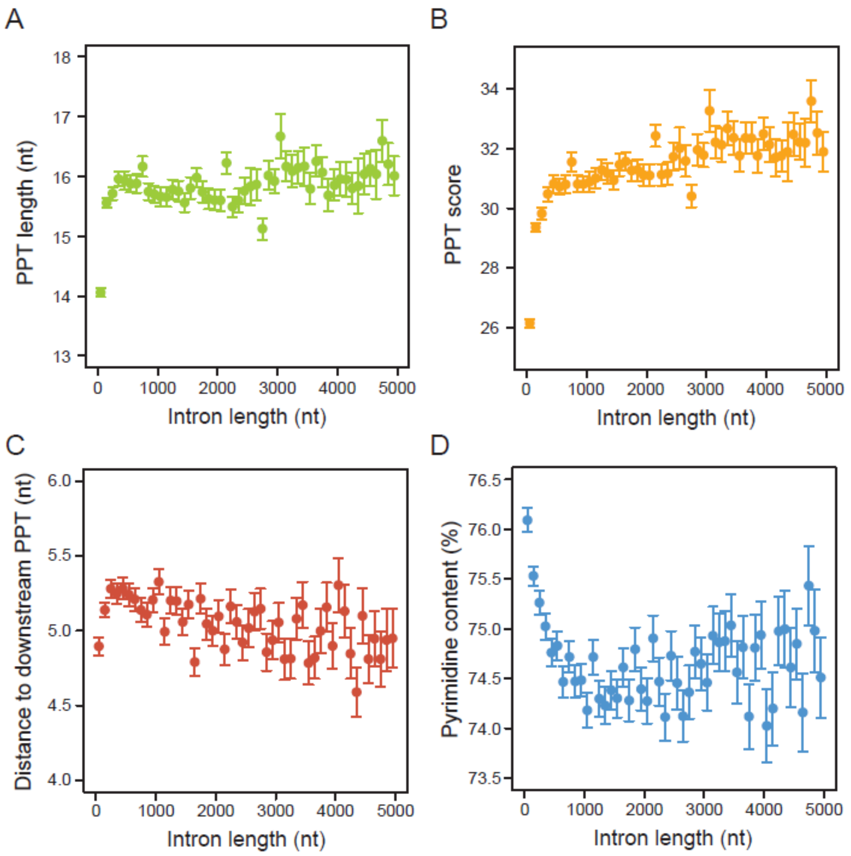

Figure S14 – PPT-associated features depending on intron length.
Mean PPT length (**A**), PPT score (**B**), distance do downstream PPT (**C**) and pyrimidine content between the BP and the 3SS (**D**) as a function of intron length. This was computed in bins of 100 nts. For visualization purposes, features are plotted for introns of length up to 5000nts. The error bars represent the standard error.

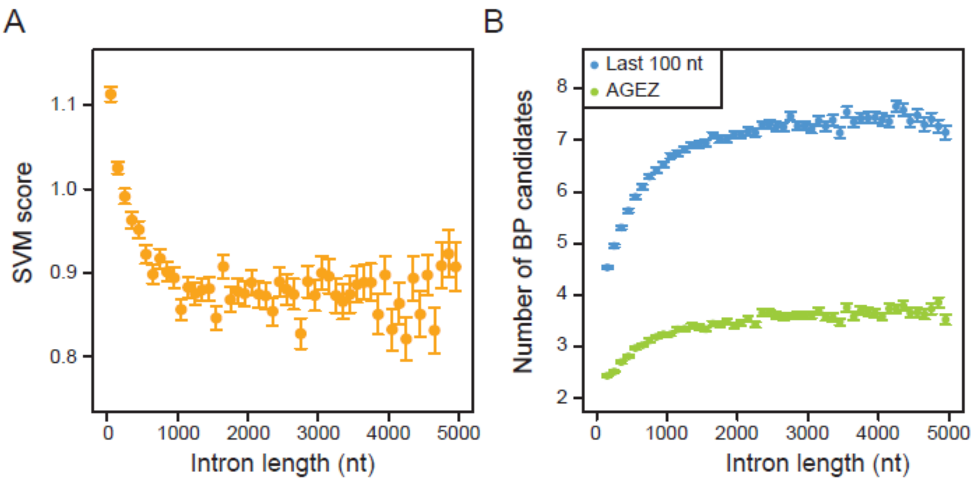

Figure S15 – Long introns have more BP candidates in the 3' end.
A – Mean BP sequence score as a function of intron length. This was computed in bins of 100 nts. **B** – Mean number of BP candidates in the 3' most 100 nt (blue) and in the AGEZ (green), depending on intron length. This was computed in bins of 100 nts. The error bars represent the standard error.

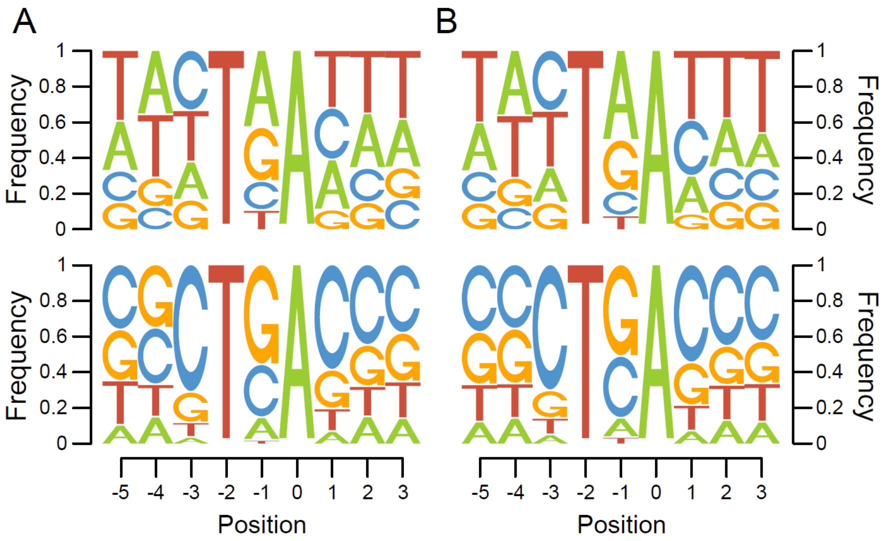

Figure S16 – Sequence logos for BPs located in GC-rich and GC-poor introns.
Sequence logos for BPs belonging to the training set (**A**) and to the predicted set (**B**), depending on intron GC-content: low (GC <= 40%; upper panels); and high (GC> 60%; lower panels).

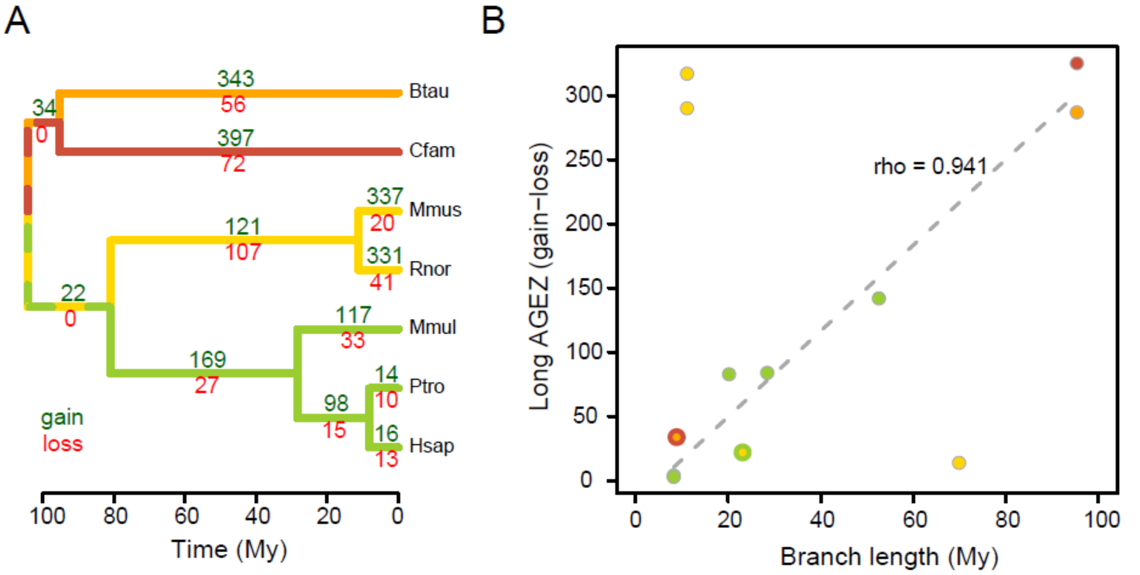

Figure S17 – Long AGEZ gain and loss in the mammalian lineage.
Using Dollo parsimony, gain (short to long AGEZ) and loss (long to short AGEZ) in each branch were computed. The initial set contained only introns for which the AGEZs were either long (p<0.01) or short (>=0.05) in the 7 species considered (N=2169). All cases in which no variation was observed, or with intermediate AGEZ sizes (0.05>p>=0.01), were discarded**. A** – Long AGEZ gain and loss for each branch. **B** – Relation between total long AGEZ gain (gain-loss) and branch length. The Spearman rank correlation rho is also shown, discarding the rodent lineage branches. Branch lengths adapted from Benton MJ, Donoghue PC. Paleontological evidence to date the tree of life. Mol Biol Evol. 2007 Jan;24(1):26-53.
